# Kinase–substrate Edge Biomarkers Provide a More Accurate Prognostic Prediction in ER-negative Breast Cancer

**DOI:** 10.1016/j.gpb.2019.11.012

**Published:** 2021-01-13

**Authors:** Yidi Sun, Chen Li, Shichao Pang, Qianlan Yao, Luonan Chen, Yixue Li, Rong Zeng

**Affiliations:** 1 CAS Key Laboratory of Systems Biology, CAS Center for Excellence in Molecular Cell Science, Institute of Biochemistry and Cell Biology, Shanghai Institutes for Biological Sciences, Chinese Academy of Sciences, Shanghai 200031, China; 2 University of Chinese Academy of Sciences, Shanghai 200031, China; 3 Deptartment of Statistics, School of Mathematical Sciences, Shanghai Jiao Tong University, Shanghai 200240, China; 4 School of Life Sciences and Biotechnology, Shanghai Jiao Tong University, Shanghai 200240, China; 5 Department of Life Sciences, ShanghaiTech University, Shanghai 201210, China; 6 CAS Center for Excellence in Animal Evolution and Genetics, Chinese Academy of Sciences, Kunming 650223, China; 7 Bio-Med Big Data Center, Key Laboratory of Computational Biology, CAS-MPG Partner Institute for Computational Biology, Shanghai Institute of Nutrition and Health, Shanghai Institutes for Biological Sciences, Chinese Academy of Sciences, Shanghai 200031, China; 8 Collaborative Innovation Center for Genetics and Development, Fudan University, Shanghai 200032, China; 9 Shanghai Center for Bioinformation Technology, Shanghai Academy of Science & Technology, Shanghai 201203, China

**Keywords:** ER-negative breast cancer, Edge biomarkers, Kinase, Substrate, Prognostic prediction

## Abstract

The estrogen receptor (ER)-negative breast cancer subtype is aggressive with few treatment options available. To identify specific prognostic factors for **ER-negative breast cancer**, this study included 705,729 and 1034 breast invasive cancer patients from the Surveillance, Epidemiology, and End Results (SEER) and The Cancer Genome Atlas (TCGA) databases, respectively. To identify key differential **kinase**–**substrate** node and **edge biomarkers** between ER-negative and ER-positive breast cancer patients, we adopted a network-based method using correlation coefficients between molecular pairs in the kinase regulatory network. Integrated analysis of the clinical and molecular data revealed the significant prognostic power of kinase–substrate node and edge features for both subtypes of breast cancer. Two promising kinase–substrate edge features, *CSNK1A1*–*NFATC3* and *SRC*–*OCLN*, were identified for more accurate **prognostic prediction** in ER-negative breast cancer patients.

## Introduction

Breast cancer is the most frequently diagnosed cancer and the leading cause of cancer mortality among females worldwide [1]. Each year, 25% of all cancer occurrences and 15% of cancer deaths among females are attributed to breast cancer [2]. Based on the presence or absence of estrogen receptor (ER), this heterogeneous disease can be divided into two subtypes. The ER-positive subtype is more common and can be treated by ER modulators, but drug resistance and relapse happen frequently in this subtype [3], [4]. The ER-negative subtype is less frequent but is more aggressive and associated with poor prognosis. To date, ER-negative patients have limited effective therapies, and chemotherapy is the mostly used treatment options [5]. Retrospective studies revealed that hormone therapies did not reduce the risk of ER-negative breast cancer [6]. Targeted therapies, such as various kinase inhibitors, offered more hope for the treatment of ER-negative breast cancer with the expression of *HER2* (Human epidermal growth factor receptor 2) [7]. A recent study reported that *PTEN* (Phosphatase and tensin homolog) loss in African American females was significantly correlated with the occurrence of ER-negative breast ductal cancer [8]. Inherited mutations in *PALB2* (Partner and localizer of *BRCA2*) and *FANCM* (Fanconi anemia complementation group M) are also found to be connected with the absence of ER [9]. These studies indicate the importance of exploring the genetic characteristics of breast cancer for the development of targeted therapies for ER-negative breast cancer. Several studies have identified differential gene expression patterns in the ER-negative subtype but are limited by relatively small-scale observational studies or particular geographic regions [10], [11], [12]. The Cancer Genome Atlas (TCGA) project has generated a substantial amount of data from a large number of patient samples [13], [14], and the clinical utility of these genomic data has been assessed in several cancer types [15], [16]. Studies based on large databases that seek to identify the potential clinical utility of molecular profiles for ER-negative breast cancer treatment are needed.

Previous studies have reported the important roles of kinases in the development of various diseases, and numerous kinases are involved in promoting cell proliferation and cancer [17]. Kinases typically contain a serine, threonine or tyrosine residue to catalyze their substrates [18]. Considering the shared conservative secondary structure element, kinases are favourable spots for targeted drugs, such as imatinib and sorafenib [19], which are effective kinase inhibitors for chronic myeloid leukaemia treatment. Although kinases are involved in many key signaling pathways in breast cancer through phosphorylating downstream substrates [12], [20], [21], [22], the interactions between kinases and substrates are also critical in biological pathways and cell signaling related to other diseases [23], [24], [25]. In other words, compared with single kinase or substrate molecule, the kinase**–**substrate network or edge interactions constituted by these molecules are considered to be more credible and permanent for characterizing complex diseases. To date, various advances have been achieved in discovering network-based biomarkers thanks to the vast accessibility of omics data [26]. A recent study reported that the prediction of multiple phenotypes has been improved based on the pathway modules constructed from the biological network [27]. Another study identified a significant association between a module enriched with cell death genes and ovarian cancer survival based on the co-expression network approach [28]. Molecular network-based markers are generally represented as the correlation coefficient between a pair of molecules, but false positives are highly likely to occur given numerous indirect associations detected by this method [29]. To solve this problem and considering the important role of kinase network in breast cancer, we mainly focused on the kinase**–**substrate interaction network in this study.

The global aim of this study was to identify differential prognostic kinase**–**substrate network biomarkers between ER-positive and ER-negative subtypes as potential drug targets for the treatment of breast cancer patients. We first analyzed the clinicopathological features and survival probabilities of ER-positive and ER-negative subtypes from the Surveillance, Epidemiology, and End Results (SEER) and TCGA databases to identify clinical prognostic factors. In addition, we selected key differential kinase**–**substrate node and edge features between the two breast cancer subtypes and integrated these selected features with clinical characteristics for prognostic prediction based on TCGA data. Moreover, we explored the possibility of kinase**–**substrate biomarkers for prognostic prediction in ER-positive and ER-negative breast invasive carcinoma.

## Results

### Clinicopathological characteristics of ER-positive and ER-negative patients

We included 705,729 SEER and 1034 TCGA breast cancer patients in this study. More than 70% of patients were ER-positive in both databases, whereas ER-negative cases accounted for 21.3% and 22.7% of patients in SEER and TCGA, respectively. As shown in **Table 1**, in SEER, ER-negative patients were diagnosed at younger ages and later disease stages with a larger proportion of African American patients compared with ER-positive patients (Chi-squared test; *P* < 0.001). In addition, ER-negative patients had a larger proportion of poorly differentiated tumors and larger tumor sizes (Chi-squared test; *P* < 0.001). In TCGA, significant differences also existed between the ER-negative and ER-positive groups, in terms of age (Chi-squared test; *P* = 0.007), race (Chi-squared test; *P* < 0.001), and lymph node status (Chi-squared test; *P* < 0.001). No significant difference in tumor stages was observed between the two subtypes, but a larger proportion of stage II patients were found for the ER-negative subtype compared with that for the ER-positive group in TCGA (62.6% *vs* 54.5%), which was consistent with that in SEER (Table 1).

**Table 1:** **Clinicopathological characteristics of ER^±^and ER^−^ breast cancer patients**

*Note*: AJCC, American Joint Committee on Cancer; ER, estrogen receptor; SEER, the Surveillance, Epidemiology, and End Results; TCGA, The Cancer Genome Atlas. Chi-squared test was used for *P* value calculation.

### Clinical prognostic factors in ER-positive and ER-negative subtypes

**Figure 1:**
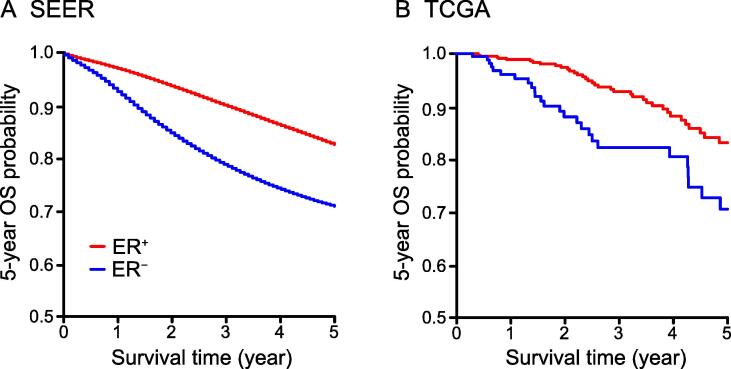
**OS probability of ER-positive and ER-negative patients** **A.** The 5-year OS probability of ER-positive and ER-negative breast cancer patients in SEER database. Log-rank test, *P* < 0.001. **B.** The 5-year OS probability of ER-positive and ER-negative breast cancer patients in TCGA database. Log-rank test, *P* = 0.02. ER, estrogen receptor. OS, overall survival; SEER, the Surveillance, Epidemiology, and End Results; TCGA, The Cancer Genome Atlas.

Patients with ER-negative breast cancer subtype exhibited a significantly lower 5-year overall survival probability in both SEER (Log-rank test; *P* < 0.001) and TCGA (Log-rank test; *P* = 0.018) datasets (Figure 1). Taking tumor stages and age groups into consideration, the ER-negative patients exhibited lower or a tendency of lower 5-year survival rates than ER-positive patients in all stages and age groups in both datasets, except the ER-negative patients aged ≥ 70 years in TCGA which showed a tendency of a higher 5-year survival rate (Figures S1 and S2; Table S1). However, the 10-year survival rate of ER-negative patients aged ≥ 70 years in TCGA was considerably reduced compared with the ER-positive patients (Figure S2). In addition, the most significant survival difference between patients with these two cancer subtypes was found for stage III (*P* < 0.001 in both SEER and TCGA, Table S1). Not surprisingly, ER-negative patients exhibited a significantly lower 10-year survival probability in the younger group of patients less than 50 years old (SEER, *P* < 0.001; TCGA, *P* = 0.049, Figure S2). For patients with positive lymph node status, absence of ER is an effective indicator of poor prognosis (SEER, *P* < 0.001; TCGA, *P* = 0.007, Figure S3).

Univariate Cox proportional hazard analysis was conducted on all the clinical factors to explore their effects on overall survival (Table S2). ER-negative patients exhibited worse survival probabilities in both the SEER and TCGA datasets [SEER, *P* < 0.001, hazard ratio (HR) = 1.372, 95% confidence interval (CI) = 1.358–1.386; TCGA, *P* = 0.021, HR = 1.642, 95% CI = 1.078–2.501]. Multivariate Cox regression survival analysis adjusted for age, race, AJCC stage, lymph node status, tumor grade, and tumor size consistently exhibited a strong correlation of the ER-negative subtype with a poor survival probability in SEER dataset (*P* < 0.001, HR = 1.356, 95% CI = 1.337–1.376). The same phenomenon also occurred in TCGA dataset (*P* = 0.002, HR = 2.170, 95% CI = 1.330–3.541) after excluding other covariates (Table S3).

### Differential kinase–substrate features between two subtypes

**Figure 2:**
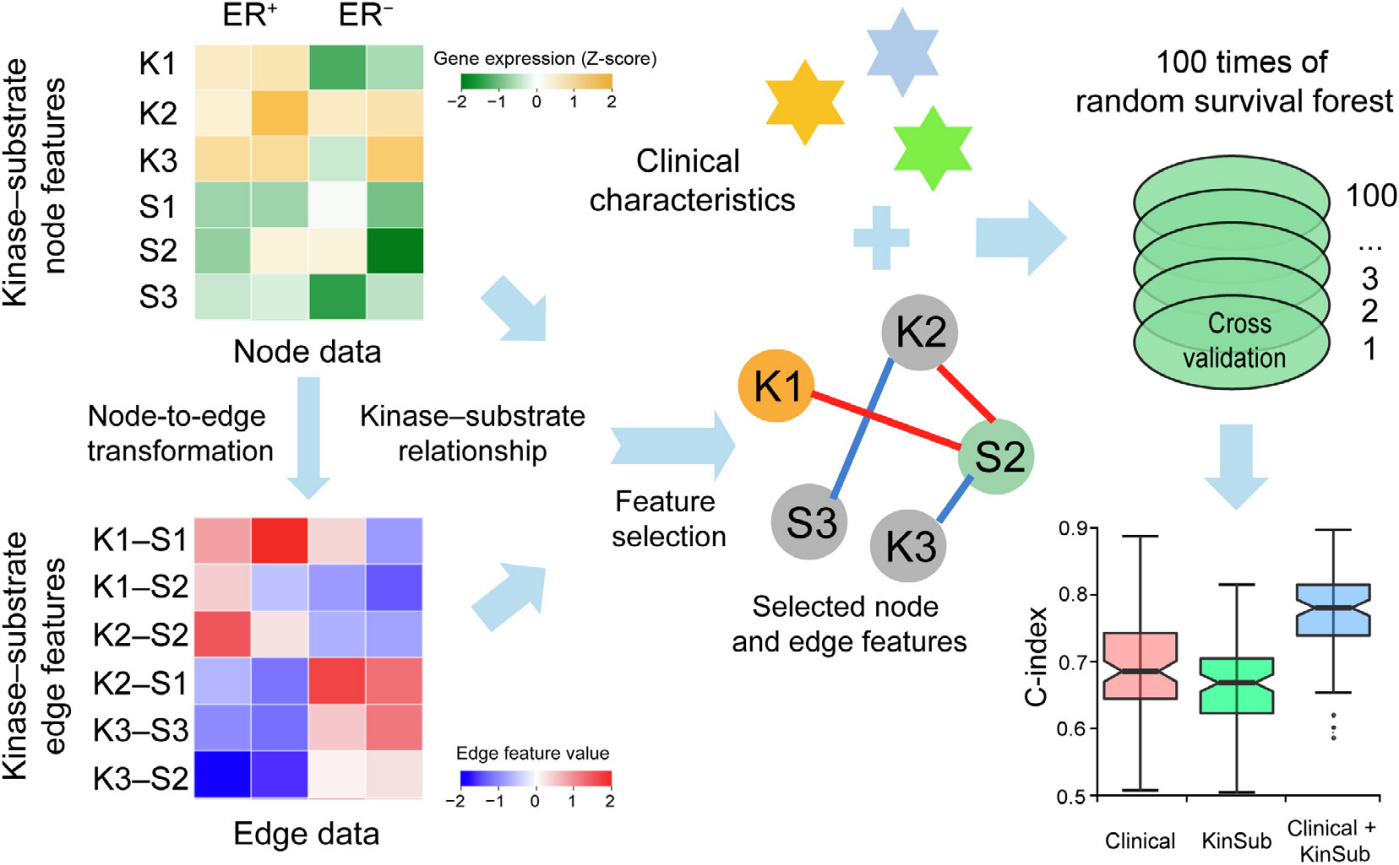
**Workflow of the kinase–substrate biomarker detection process** Gene expression levels (Z-score transformed) are presented in a green-yellow color gradient with green and yellow for low and high expression, respectively. Features in the node data matrix are transformed into edge data based on correlation between each pair of kinase and substrate, generating the edge dataset in a blue-red color gradient with blue and red for low and high correlation, respectively. “K” and “S” depict kinase and substrate, respectively, and numbers 1–3 indicate different kinases and substrates, *e.g*., “K1” means “Kinase1” and “S1” means “Substrate1”. The yellow and green colored “K1” and “S2” indicate the differentially expressed kinase and substrate, respectively. The red and blue lines delineate the positive and negative correlations, respectively, between the pair of kinase and substrate. Node and edge features are subsequently selected using the LASSO regression algorithm, and 100 times of Monte Carlo cross validation are performed to identify the prognostic value of these selected features by integrating clinical characteristics for prognostic prediction. Clinical: clinical variables only; KinSub, kinase–substrate node and edge features only; Clinical + KinSub, clinical plus kinase–substrate node and edge features.

The kinases included in this study comprised 470 genes annotated in the UniProtKB/Swiss-Prot database [30]. Experimentally validated substrates of these kinases from PhosphositePlus [31] were also incorporated. Kinase**–**substrate edge features were constructed based on the method described previously [24]. The kinase–substrate node features were transformed into kinase**–**substrate edge features according to the correlation of each kinase**–**substrate pair (see Method). We subsequently conducted feature selection of these node and edge features between ER-positive and ER-negative subtypes. By using 100 times of Monte Carlo cross validation, the selected kinase**–**substrate node and edge features were integrated with clinical characteristics for prognostic prediction (Figure 2). The clinical characteristics reported here included age, race, tumor stage, and lymph node status, all of which exhibited significant differences between ER-positive and ER-negative subtypes (Table 1).

**Figure 3:**
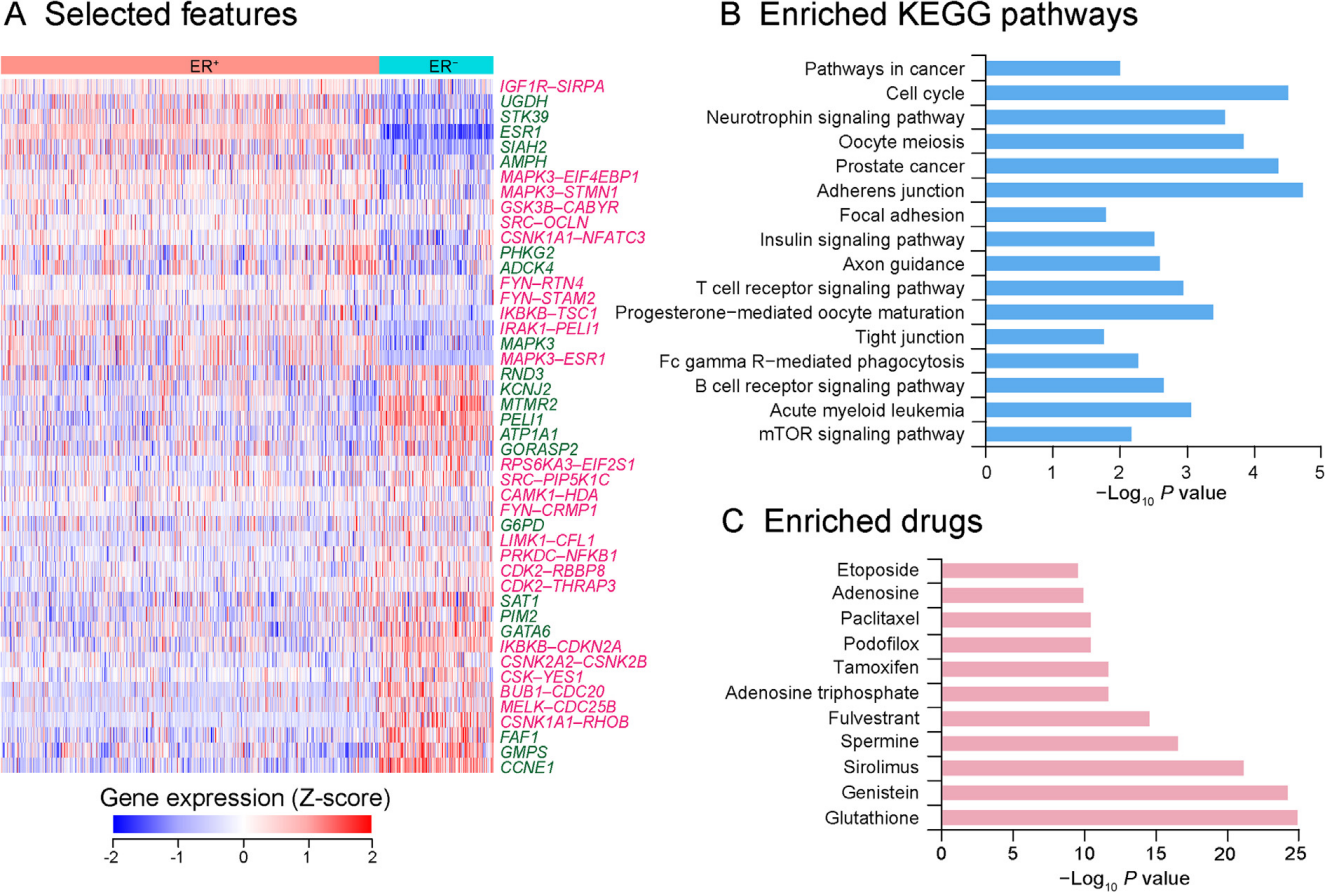
**Features selectedfrom LASSO regression** **A.** Heatmap demonstrating the Z-score transformed expression levels of the 21 differential node features and 25 differential edge features between ER-positive and ER-negative subtypes. Blue and red represent low and high expression, respectively. Green and pink texts indicate node and edge features, respectively. **B.** KEGG-enriched pathways of the 46 selected node and edge features. **C.** Drugs enriched for the 46 selected node and edge features. KEGG, Kyoto Encyclopedia of Genes and Genomes.

By using the least absolute shrinkage and selection operator (LASSO) [32], a tatol of 46 differential kinase**–**substrate node and edge features between ER-positive and ER-negative subtypes were identified from the molecular dataset (Figure 3A). More than half of the selected features were upregulated in the ER-negative subtype, while *ESR1* and *MAPK3* were highly expressed in ER-positive subtype, which was consistent with the previous report [13]. Five-fold cross validation was performed during the process to tune the value of lambda in LASSO, and the performance was evaluated by the area under the curve (AUC), which was 0.908 (Figure S4, see Method). Kyoto Encyclopedia of Genes and Genomes (KEGG) pathway enrichment analysis was conducted on the selected node and edge features, and most of them were highly enriched in cell cycle and cancer-related pathways (Figure 3B). Moreover, by analyzing the associated drugs of these features, we found that they could be highly enriched in existing drugs, such as glutathione and genistein (Figure 3C).

### Kinase–substrate node and edge features improve prognostic prediction

**Figure 4:**
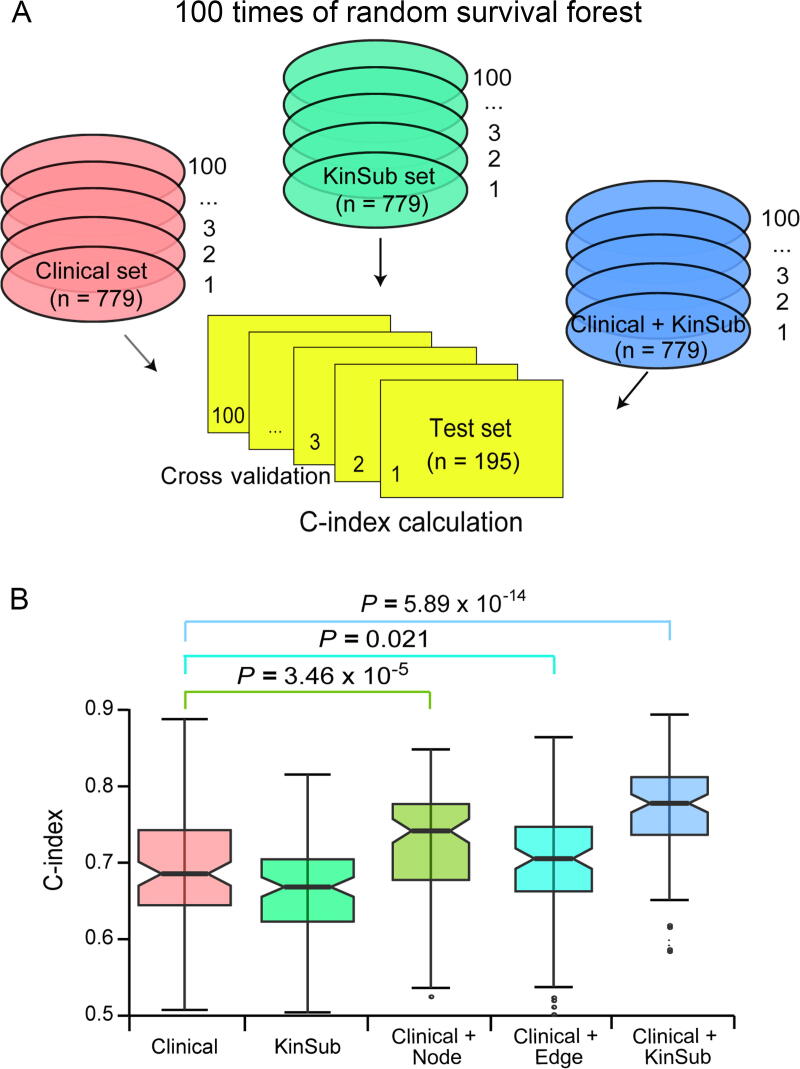
**Random survival forest models trained from clinical variables and kinase**–**substrate node and edge features** **A.** 100 times of five-fold cross validation in the clinical, KinSub, and their combined datasets for C-index calculation. **B.** Comparison of C-indexes of 100 times of cross validation results for Clinical, KinSub, Clinical plus KinSub node features (Clinical + Node), Cnical plus KinSub edge features (Clinical + Edge), and Clinical + KinSub. n, the number of samples. Two-sided Wilcoxon rank-sum test was used for significance test.

To assess whether these kinase and substrate features can provide additional prognostic power compared with clinical variables, we built predictive models by integrating clinical variables with expression values of the selected node and edge features. Concordance index (C-index) was used to measure the predictive power of node and edge features together with clinical variables, and a C-index greater than 0.5 indicates prediction accuracy other than random guess [33]. We applied 100 times of five-fold and two-fold cross validation for each model and calculated 100 C-indexes for each group of predictive variables (Figure 4, Figure S5). Notably, models integrating clinical variables with either node features (“Clinical + Node” model) or edge features (“Clinical + Edge” model) significantly increased the predictive accuracy compared with the model based exclusively on clinical variables (“Clinical” model) (C-index 0.744 *vs*. 0.683, *P* = 3.46 × 10^−5^; C-index 0.708 *vs*. 0.683, *P* = 0.021; Figure 4B). The final model integrating clinical variables with all the kinase–substrate node and edge features (“Clinical + KinSub” model) demonstrated the highest prediction power (C-index 0.781 *vs*. 0.683, *P* = 5.89 × 10^−14^; Figure 4B). Moreover, we retrieved the expression of a 50-gene qPCR assay (PAM50) gene set from our dataset and built a prognostic model by integrating clinical variables with “PAM50” (“Clinical + PAM50” model) using the same method. PAM50 gene signatures are widely used intrinsic subtype markers in breast cancer with independent prognostic values [34], but surprisingly, the “Clinical + PAM50” model performed no better than the “Clinical + KinSub” model in our analysis (C-index 0.743 *vs*. 0.781, *P* value = 2.73 × 10^−4^; Figure S6), implying the better prognostic potential of kinase–substrate node and edge features in breast cancer.

### Kinase–substrate biomarkers exhibit subtype-specific prognostic power

To identify subtype-specific biomarkers for prognostic prediction, we performed univariate survival analysis for each of the 46 molecular features in both ER-positive and ER-negative subtypes (Table 2). Four node features *SAT1* (*P* = 0.027, HR = 0.539, 95% CI = 0.312–0.931), *GMPS* (*P* = 0.011, HR = 1.895, 95% CI 1.158–3.101), *PHKG2* (*P* = 0.005, HR = 0.491, 95% CI = 0.297–0.811), *CCNE1* (*P* = 0.016, HR = 1.833, 95% CI = 1.122–2.995) and one edge feature *BUB1*–*CDC20* (*P* = 0.013, HR = 1.867, 95% CI = 1.139–3.063) demonstrated significantly prognostic power in ER-positive subtype. Two edge features *CSNK1A1*–*NFATC3* (*P* = 0.043, HR = 2.048, 95% CI = 1.021–4.107) and *SRC***–***OCLN* (*P* = 0.048, HR = 2.04, 95% CI 1.006–4.134) showed significantly prognostic power in the ER-negative group. To exclude the influence of clinical covariates, a multivariate Cox model was constructed and the prognostic values of these candidate biomarkers were validated (Table S4).

**Table 2:** **Univariate survival analysis of 46 kinase–substratenode and edge biomarkers in breast cancer based on ER statuses**

*Note*: CI, confidence interval; HR, hazard ratio. Wald test was used for *P* value calculation.

**Figure 5:**
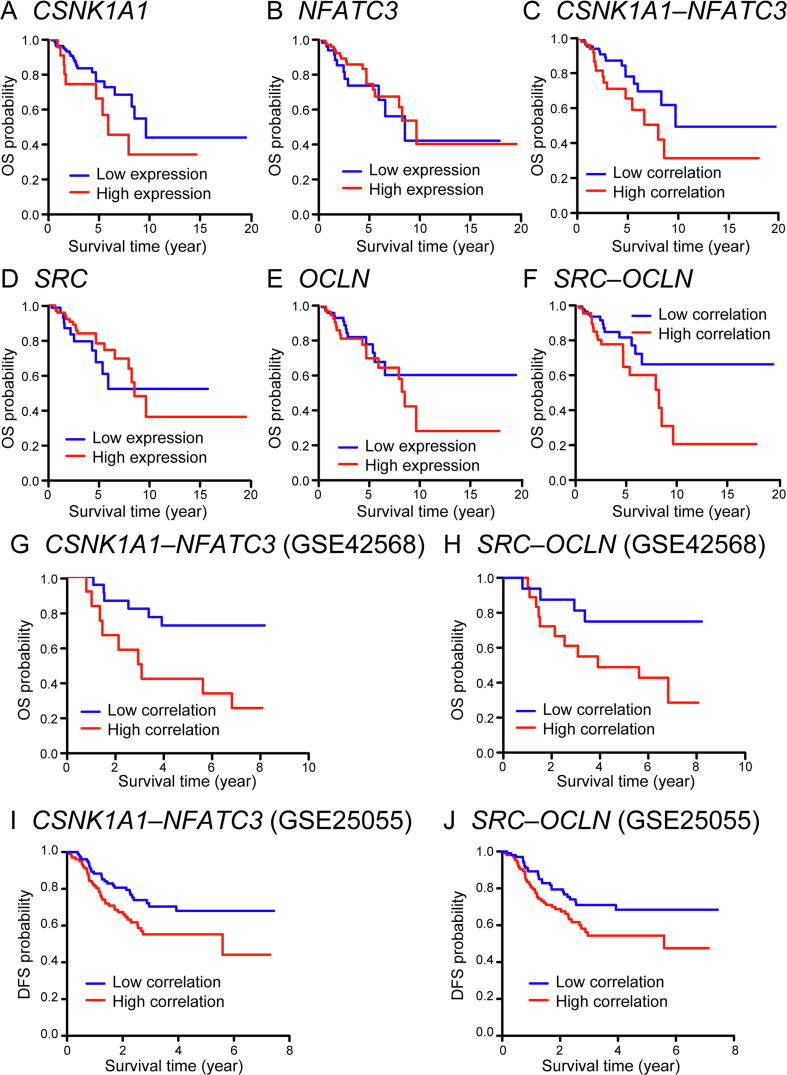
**Prognostic values of two edge biomarkers in ER-negative patients** **A.** Kaplan–Meier curves of high and low expression groups stratified by *CSNK1A1* for ER-negative breast cancer patients in TCGA. Log-rank test, *P* = 0.111. **B.** Kaplan–Meier curves of high and low expression groups stratified by *NFATC3* for ER-negative breast cancer patients in TCGA. Log-rank test, *P* = 0.418. **C.** Kaplan–Meier curves of high and low correlation groups stratified by *CSNK1A1*–*NFATC3* for ER-negative breast cancer patients in TCGA. Log-rank test, *P* = 0.039. **D.** Kaplan–Meier curves of high and low expression groups stratified by *SRC* for ER-negative breast cancer patients in TCGA. Log-rank test, *P* = 0.476. **E.** Kaplan–Meier curves of high and low expression groups stratified by *OCLN* for ER-negative breast cancer patients in TCGA. Log-rank test, *P* = 0.378. **F.** Kaplan–Meier curves of high and low correlation groups stratified by *SRC*−*OCLN* for ER-negative breast cancer patients in TCGA. Log-rank test, *P* = 0.043. **G.** Kaplan–Meier curves of high and low correlation groups stratified by *CSNK1A1*–*NFATC3* in the GSE42568 dataset. Log-rank test, *P* = 0.01. **H.** Kaplan–Meier curves of high and low correlation groups stratified by *SRC*–*OCLN* in the GSE42568 dataset. Log-rank test, *P* = 0.036. **I.** Kaplan–Meier curves of high and low correlation groups stratified by *CSNK1A1*–*NFATC3* in the GSE25055 dataset. Log-rank test, *P* = 0.022. **J.** Kaplan–Meier curves of high and low correlation groups stratified by *SRC*–*OCLN* in the GSE25055 dataset. Log-rank test, *P* = 0.033. DFS, disease-free survival.

To further assess the relationship of these candidate biomarkers with clinical outcome of ER-positive and ER-negative patients, Kaplan–Meier curves were constructed using Log-rank test to stratify the patients into high- and low-risk groups according to the expression levels of node features or correlation values of edge features (median split) (Figure 5, Figure S7). Poor survival was observed in the high-risk groups of ER-negative patients stratified by *CSNK1A1*–*NFATC3* (Figure 5C) and *SRC*–*OCLN* (Figure 5F). Moreover, Kaplan–Meier curves were also plotted based on the expression of *CSNK1A1*, *NFATC3*, *SRC*, and *OCLN* by median split (Figure 5A, B, D, and E), but the expression of these kinases and substrates did not demonstrate prognostic values in ER-negative patients, which supports the significant power of the correlations between kinases and substrates in clinical practice. Independent datasets from Gene Expression Omnibus (GEO), including GSE42568 (HG-U133A Plus2 platform) [35], GSE22055 (HG-U133A platform) [36], and ten other datasets with available survival information and ER statuses, were used to determine whether the identified node and edge biomarkers could provide prognostic information for ER-positive and ER-negative patients. We observed that these potential biomarkers could also stratify the survival of high- and low-risk groups in these independent datasets, suggesting that the prognostic power of these biomarkers is stable and reliable in practice (Figure 5G–J, Figure S7; Table S5).

We next compared the kinase**–**substrate biomarkers with existing breast cancer prognostic biomarkers from previous studies [12], [37]. National Research Council (NRC) gene signatures NRC-1 (33 genes), NRC-2 (46 genes), and NRC-3 (47 genes) were reported to predict disease-free survival of ER-positive patients with high accuracy. NRC-7, NRC-8, and NRC-9 gene sets (39, 25, and 20 genes, respectively) were prognostic signatures for ER-negative subtype. We compared the performance of our newly identified biomarkers with the previously reported signatures by C-indexes based on 100 times of cross validation in the two subtypes, respectively (see Method). For the ER-positive biomarkers, *CCNE1* and the combination of the five biomarkers demonstrated higher prognostic effects than both NRC-1 and NRC-2 (Figure S8A). For the ER-negative biomarkers, *SRC*–*OCLN*, *CSNK1A1*–*NFATC3*, and their combination significantly outperformed NRC-7, NRC-8, NRC-9 and their combination, as well as “S6 kinase” markers, which include genes encoding kinases in the S6 kinase signaling pathway, such as *RPS6KA3*, *SMG-1*, and *RPS6KA1* [12] (Figure S8B). These results suggested that the novel kinase**–**substrate biomarkers identified in this study had better performance than existing biomarkers, which were gene sets comprising dozens of genes.

To demonstrate the additional prognostic power of clinical characteristics from the previous analysis, we also explored the combinations of these node and edge biomarkers with clinical factors. The C-index and *P* value from the Cox regression model were presented for all possible combinations. We found that the inclusion of age group, AJCC stage, and lymph node status in ER-positive or ER-negative subtype could significantly improve the prognostic power (Table S6). Kaplan–Meier survival curves also demonstrated that combination of clinical factors with these biomarkers achieved increased prognostic power (Figure 5C and F, Figures S7A and S9).

To evaluate the potential for specific targeted drug development for these node and edge biomarkers, we compared the expression of the selected kinases and substrates in ER-positive and ER-negative patients with that in normal individuals in the TCGA database. The expression of most of the selected kinases and substrates was increased in the breast cancer compared with that in normal samples (Figure S10). Specifically, the expression level of SRC was also higher in the ER-negative group than that in the ER-positive subtype (Figure S10). Considering the poor survival of high-risk groups of ER-negative patients stratified by *SRC*–*OCLN*, the edge biomarkers could serve as potential drug targets; however, further studies are needed.

## Discussion

This study analyzed 706,763 first primary invasive breast cancer patients with available ER status from two independent databases (SEER and TCGA). This study was the first to integrate clinical factors with kinase**–**substrate node and edge biomarkers for prognostic prediction between ER-positive and ER-negative breast cancer subtypes in large datasets. In addition, we identified prominent kinase**–**substrate node and edge biomarkers in both subtypes, and these signatures might be potential biomarkers to distinguish ER-positive and ER-negative breast cancer.

The data presented here confirmed that the ER-negative subtype exhibited poorer survival with an increased occurrence rate among younger ages and patients of African American compared with ER-positive patients. This finding was consistent with the phenomenon observed in the California dataset [38], [39], [40]. In addition, we found that features including later disease stages and larger tumor sizes were associated with the ER-negative subtype. These observations were reasonable given the lower survival rate of ER-negative patients. Interestingly, in both datasets, patients in the 50–69-year-old group exhibit the largest proportion of ER-negative breast cancer occurrence (Table 1), and the survival probability of this group was similar in SEER or even higher in TCGA compared with patients less than 50 years old (Figure S2). This finding might be related to hormone levels in the body. Carey et al. [38] reported the increased prevalence of the more aggressive subtype in premenopausal women compared with postmenopausal women in the Carolina breast cancer cohort. Further studies are needed to better characterize the influence of hormone level on the occurrence of breast cancer subtypes.

We identified several key distinct kinase–substrate node and edge features between ER-positive and ER-negative subtypes. By analyzing the associated drugs of these features, we found that they could be highly enriched in existing drugs, such as glutathione and genistein. Glutathione is involved in numerous biological processes, including but not limited to cell development, differentiation, antioxidation, and immune response modulation. Therefore, disorders in glutathione metabolism could lead to the development and progression of numerous human diseases, including cancer [41], [42], [43]. In fact, as a drug the glutathione has been used in the treatment of lung cancer and liver cancer [44], [45], [46], [47], [48], implying that it could potentially be used in the treatment of ER-negative invasive breast cancer.

Integrated analysis of molecular biomarkers with clinical prognostic factors in breast cancer patients demonstrated the utility of the inclusion of kinase**–**substrate node and edge biomarkers for prognostic prediction. Neither of the clinical plus nodes (“Clinical + Node” model) and clinical plus edges (“Clinical + Edge” model) exhibited increased prognostic power compared with the integrated prediction model (“Clinical + KinSub” model), suggesting that both the expression of kinases or substrates (nodes) and the correlation between kinases and their targeted substrates (edges) play important roles in the regulation of networks in our body. Moreover, the “Clinical + KinSub” model achieved better performance than the model built by the widely used “PAM50” gene set. The result confirmed the importance of kinase**–**substrate network in our body. In addition, the kinase**–**substrate node and edge biomarkers identified in both ER-positive and ER-negative subtypes outperformed the existing markers of breast cancer in prognosis. Considering the feasibility and convenience of kinases to be drug targets, our analysis provides a prominent kinase–substrate set for the drug development, which will give more available intervention on patients with ER-negative breast cancer in the future.

The improved prognostic value of the kinase**–**substrate edge biomarkers validated the utility of our method for identifying the correlation between genes instead of DEGs as functional drivers. These edge markers are generally missed by traditional methods [24], [49]. The disruption of the correlations between kinases and substrates can potentially improve the clinical outcome of breast cancer patients, which enlarges the scope of drug target development. The results from 12 independent GEO datasets also confirmed the effectiveness of these biomarkers (Figure 5, Figure S7; Table S5). Besides, in the two different subtypes, node features contributed more to the prognostic probability of ER-positive patients, whereas ER-negative subtype mostly relied on the edge biomarkers (Table 2). This phenomenon may underpin different regulation networks in different types of diseases. Previous studies also reported different results. Specifically, edge biomarkers were considered to be more reliable for subtyping in one study [24], whereas Speers et al. [12] demonstrated that kinases alone were effective in ER-negative breast cancer subtyping. Given the different cohorts and analyzing methods used in these studies, a more comprehensive dataset with broader networks in addition to kinase**–**substrate networks is needed in the future to testify the usefulness of these two types of biomarkers.

Particularly, two kinase**–**substrate pairs*, CSNK1A1*–*NFATC3* and *SRC*–*OCLN*, demonstrated strong correlations with clinical outcome in the ER-negative subtype. *NFATC3* is one member of the NFAT (nuclear factor of activated T cells) transcription factor gene family, which play important roles in T cell activation [50]. The activation of calcineurin-NFAT pathway was observed in triple-negative breast cancer and substantially contributed to the tumorigenesis and metastasis of mammary tumor cell lines [51], [52], [53]. In our study, the high correlation between *CSNK1A1* and *NFATC3* is associated with poor survival in the ER-negative group, which suggests that pharmacological inhibition of *NFATC3* by targeting *CSNK1A1* could be of therapeutic interest for breast cancer patients. The tight junction structure is one of the inevitable barrier for cancer cells to enable metastasis, whereas OCLN (Occludin) is one of the early identified tight junction proteins [54], [55], [56]. Slight association between reduced *OCLN* expression and poor overall survival was observed in a cohort with 10-year follow-up of breast cancer patients, and studies conducted on human cell lines demonstrated that OCLN phosphorylation by SRC attenuates its assembly at the tight junctions [56], [57], [58], [59]. Given that the high correlation group between *SRC* and *OCLN* had worse survival performance in ER-negative subtype, the association between *SRC* and *OCLN* represents a potential “driver” of cell proliferation in ER-negative breast cancer.

In conclusion, our study depicts a model for the identification of promising molecular biomarkers with utility in clinical prognosis. This population-based research suggests distinct clinicopathological characteristics between ER-positive and ER-negative breast cancer patients. Prognostic clinical factors and kinase**–**substrate node and edge features were identified based on the comparison of these two subtypes. Compared with using the clinical variables only ("Clinical" model), incorporating kinase**–**substrate node and edge features greatly improves the predictive accuracy, indicating the advantages of kinases and substrates as well as their regulation in clinical diagnosis. Furthermore, our analyses also provide promising kinase**–**substrate node and edge biomarkers for clinically relevant refinement of prognostic assessment in the ER-positive and ER-negative subtypes, and these biomarkers also serve as candidate drug targets for the treatment of breast invasive cancer in the future. In addition, this work can be applied to the analyses of network biomarkers [24], [49], [60], [61], [62], [63] and dynamic network biomarkers [25], [64], [65], [66] for disease diagnosis and disease prediction, respectively.

## Method

### Clinical database

The SEER 1973 to 2012 database (http://seer.cancer.gov/about/overview.html) represented approximately 28% of the US population. We analyzed breast cancer survival in all women diagnosed with first primary breast cancer with ER status (available from 1990). Characteristics, including age at diagnosis, race, AJCC stage, lymph node status, tumor grade, and tumor size, were examined for each patient. Survival information included vital status, cause of death, and survival time. All characteristics studied and information regarding ER status were based on standard coding rules of SEER records. After excluding patients without survival information, the final study cohort of SEER was reduced to 705,729 from an initial dataset of 705,740.

Clinical data of breast invasive carcinoma from TCGA were used for this study (http://gdac.broadinstitute.org/runs/stddata__2016_01_28/data/BRCA/20160128). The clinicopathological information for each patient included age at diagnosis, race, AJCC stage, and lymph node status. ER status was determined according to the current clinical guideline jointly issued by the American Society of Clinical Oncology (ASCO) and the College of American Pathology [67]. After excluding male patients and cases lacking information on ER status, the final study cohort was reduced to 1034 from an initial dataset of 1097.

### Gene expression dataset

Analysis of kinase**–**substrate features was performed on gene expression data (RNAseqV2) of TCGA Breast Invasive Carcinoma (BRCA) (http://gdac.broadinstitute.org/runs/stddata__2016_01_28/data/BRCA/20160128). Upper quartile normalized RNA-seq by Expectation-Maximization (RSEM) data were log_2_ transformed, and the data were then Z-score centred on the gene level [68].

Gene expression data were matched with clinical data using a TCGA barcode for each patient, excluding cases lacking clinical records or expression information, which resulted in a cohort of 1017 breast invasive cancer patients. In total, 470 of 521 known human kinases and 552 experimentally validated substrates of these kinases were identified in the expression dataset and characterized as node features [30].

All the GEO datasets were obtained from the GEO website (GEO: GSE42568, GSE22055, GSE10893, GSE2034, GSE21653, GSE22133, GSE22219, GSE48408, GSE4922, GSE53031, GSE6532, and GSE7390), and are publicly accessible at https://www.ncbi.nlm.nih.gov/geo.

### Kinase–substrate edge construction

Kinase–substrate node features were transformed into kinase**–**substrate edge features based on the correlation of each kinase**–**substrate pair, performed according to the method previously described [24]. The transformation is described below.

kinase,usubstrate,vxu,j,kxv,j,k->edge<u-v>kxu,j,k-μu,kσu,k.xv,j,k-μv,kσv,k

where $x_{u,j,k}$ represents the original value of u^th^ kinase in j^th^ sample from k^th^ class, $x_{v,j,k}$ represents the original value of v^th^ substrate in j^th^ sample from k^th^ class, and k was set to 1 or 2 to represent the ER-positive or ER-negative subtype. In addition, $\mu_{u,k}=\frac{1}{n_{k}}\sum_{j=1}^{n_{k}}\left(x_{u,j,k}-\mu_{u,k}\right)$ and $\mu_{v,k}=\frac{1}{n_{k}}\sum_{j=1}^{n_{k}}\left(x_{v,j,k}-\mu_{v,k}\right)$ are sample means of kinase u and substrate v, and $\sigma_{u,k}=\sqrt{\frac{1}{n_{k}}}\sum_{j=1}^{n_{k}}{\left(x_{u,j,k}-\mu_{u,k}\right)}^{2}$ and $\sigma_{v,k}=\sqrt{\frac{1}{n_{k}}}\sum_{j=1}^{n_{k}}{\left(x_{v,j,k}-\mu_{v,k}\right)}^{2}$ are the corresponding uncorrected sample standard deviation. After edge transformation, the final expression dataset consisted of 1022 kinase**–**substrate node features and 2606 edge features.

### Feature selection and performance comparison

We first selected the node and edge features using the Student’s *t*-test with a *P* value cut-off of 0.05 between ER-positive and ER-negative subtypes, which reduced the dataset to 2275 features. We used these expression data as the explanatory variables and two subtypes as the response variables to build a binary classifier (family = “binomial”) for feature selection by five-fold cross validation. For each of the five iterations, 80% of the data were used for training by LASSO using the R package “glmnet” [69], and the prediction was conducted on the remaining 20% of the data. The prediction results from the five-fold cross validation were combined, and the AUC was calculated by the R package “ROCR”. Ultimately, we trained the entire dataset with LASSO, and all the 46 features with non-zero coefficients were retained for subsequent analysis.

Clinical data, including ER status, age, AJCC stage, and lymph node status, were combined with molecular data that included the 46 selected features for model training by random survival forest (RSF) using the R package “randomForestSRC” [70]. For each dataset, we used two criteria to randomly split the samples into two parts. One method used 80% as the training set and the remaining 20% as test set. The other method divided the entire set in half: 50% served as the training set and 50% served as the test set. The model built based on the training set was then applied to the test set for prediction, and the C-index was calculated using the R package “survcomp”. For each dataset, the procedure was iterated 100 times; thus, 100 C-indexes were obtained. Wilcoxon rank-sum test was then used to compare the results from different datasets. Furthermore, each of the 46 node and edge features was evaluated for prognostic values in ER-positive and ER-negative subtypes using univariate Cox regression. For features demonstrating significant prognostic power, multivariate survival analysis was also conducted to exclude the influence of covariates.

For the performance comparison of the identified kinase**–**substrate biomarkers in ER-positive and ER-negative subtypes with existing biomarkers, we built RSF models for each biomarker and their combinations by five-fold cross validation. By iterating 100 times of the procedure, we compared the C-indexes between kinase**–**substrate biomarkers and existing biomarkers by Wilcoxon rank-sum test.

### Functional analysis

We performed KEGG pathway and drug association enrichment analysis for the 46 kinase–substrate features using WebGestalt (http://www.webgestalt.org/). Hypergeometric test was used for enrichment evaluation analysis, and the Benjamini & Hochberg method was used for *P* value adjustment. *P* values less than 0.05 were considered significant.

### Statistical analysis

R version 3.2.2 (http://www.R-project.org/) was used to perform all the statistical analyses in this work. The relationships of ER-positive and ER-negative groups with clinicopathological characteristics were analyzed using the Chi-square (χ2) test. Survival curves were generated using the Kaplan–Meier method, and the Log-rank test was applied to calculate differences between the curves. HRs and their 95% CI were estimated for each multivariate and univariate survival analyses using Cox proportional hazards models. All tests conducted were two-sided, and significant differences were noted by *P* values less than 0.05.

## CRediT author statement

**Yidi Sun:** Conceptualization, Methodology, Software, Formal analysis, Visualization, Writing - original draft. **Chen Li:** Conceptualization, Writing - review & editing. **Shichao Pang:** Writing - review & editing. **Qianlan Yao:** Writing - review & editing. **Luonan Chen:** Supervision, Writing - review & editing. **Yixue Li:** Supervision, Writing - review & editing. **Rong Zeng:** Conceptualization, Supervision, Writing - review & editing. All authors read and approved the final manuscript.

## Competing interests

The authors declare no competing financial interests.

## Supplementary material

Supplementary Figure S1 **OS probability of breast cancer patients at different stages**. The 10-year OS probability of ER-positive and ER-negative patients with stages I+II (SEER, *P* < 0.001; TCGA, *P* = 0.326; Log-rank test) (**A**) or stages III+IV (SEER, *P* < 0.001; TCGA, *P* < 0.001; Log-rank test) (**B**). OS, overall survival.

Supplementary Figure S2 **OS probability of breast cancer patients in different age groups**. The 10-year OS probability of ER-positive and ER-negative patients aged <50 years (SEER, *P* < 0.001; TCGA, *P* = 0.049; Log-rank test) (**A**), 50–69 years (SEER, *P* < 0.001; TCGA, *P* = 0.227; Log-rank test) (**B**), and ≥ 70 years (SEER, *P* < 0.001; TCGA, *P* = 0.120; Log-rank test) (**C**).

Supplementary Figure S3 **OS probability of breast cancer patients with different lymph node status**. The OS probability of ER-positive and ER-negative patients with positive lymph node status (SEER, *P* < 0.001; TCGA, *P* = 0.007; Log-rank test) (**A**), or negative lymph node status (SEER, *P* < 0.001; TCGA, *P* = 0.778; Log-rank test) (**B**).

Supplementary Figure S4 **ROC curve of the binary classifier against ER status trained with LASSO through five-fold cross validation**.

Supplementary Figure S5 **Random survival forest models trained from clinical variables and kinase–substrate node and edge features with two-fold cross validation**.

Supplementary Figure S6 **C-indexes of 100 times of cross validation of random survival forest models in TCGA dataset**. PAM50, 50-gene qPCR assay, which was used to define four intrinsic subtypes of breast cancer.

Supplementary Figure S7 **Prognostic values of node and edge biomarkers in ER-positive patients A**. Kaplan–Meier curves of ER-positive breast cancer patients indicating the prognostic values of the five biomarkers in TCGA cohort. Log-rank test; *P* = 0.024 for *SAT1*, *P* = 0.01 for *GMPS*, *P* = 0.005 for *PHKG2*, *P* = 0.014 for *CCNE1*, and *P* = 0.012 for *BUB1*–*CDC20*. **B**. Kaplan–Meier curves of ER-positive breast cancer patients indicating the prognostic values of the five biomarkers in GSE42568 dataset. Log-rank test; *P* = 0.057 for *SAT1*, *P* = 0.107 for *GMPS*, *P* = 0.22 for *PHKG2*, *P* = 0.001 for *CCNE1*, and *P* = 0.162 for *BUB1*–*CDC20*. **C**. Kaplan–Meier curves of ER-positive breast cancer patients indicating the prognostic values of the five biomarkers in GSE25055 dataset. Log-rank test; *P* = 0.015 for *SAT1*, *P* = 0.015 for *GMPS*, *P* = 0.225 for *PHKG2*, *P* < 0.001 for *CCNE1*, and *P* = 0.016 for *BUB1*–*CDC20*. DFS, disease-free survival.

Supplementary Figure S8 **Comparison of the kinase–substrate biomarkers with existing biomarkers from previous studies**. **A**. Comparison of prognostic values of biomarkers identified in this study for ER-positive breast cancer and NRC-1, NRC-2, and NRC-3 from Li and colleagues [37]. Combined, the combination of *SAT1*, *GMPS*, *PHKG2*, *CCNE*1 and *BUB1*–*CDC20*. **B**. Comparison of prognostic values of biomarkers identified in this study for ER-negative breast cancer and NRC-7, NRC-8, and NRC-9 from Li et al. [37] and the “S6 kinase” markers from Speers and colleagues [12]. Combined, the combination of *SRC*–*OCLN* and *CSNK1A1*–*NFATC3*. NRC, National Research Council gene signatures.

Supplementary Figure S9 **Prognostic power integrating the kinase–substrate biomarkers with age group, AJCC stage, or lymph node status**. **A**. Kaplan–Meier curves of ER-positive breast cancer patients indicating the prognostic power of ER-positive biomarkers combined with age groups. *P* = 0.01 for *SAT1* + age, *P* < 0.001 for *GMPS* + age, *P* = 0.005 for *PHKG2* + age, *P* < 0.001 for *CCNE1* + age, and *P* = 0.012 for *BUB1*–*CDC20* + age. **B**. Kaplan–Meier curves of ER-negative breast cancer patients indicating the prognostic power of ER-negative biomarkers combined with AJCC stages. *P* < 0.001 for *CSNK1A1*–*NFATC3*+ stage and *P* < 0.001 for *SRC*–*OCLN*+ stage. **C**. Kaplan–Meier curves of ER-negative breast cancer patients indicating the prognostic power of ER-negative biomarkers combined with lymph node statuses. *P* < 0.001 for *CSNK1A1*–*NFATC3* + lymph node status and *P* < 0.001 for *SRC*–*OCLN* + lymph node status. *P* values were calculated by Log-rank test.

Supplementary Figure S10 **Expression of the kinases and substrates constructing the ER-positive and ER-negative biomarkers**. **A**. Expression of kinases and substrates of ER-positive biomarkers in normal, ER-positive, and ER-negative groups in the TCGA cohort. **B**. Expression of kinases and substrates of ER-negative biomarkers in normal, ER-positive, and ER-negative groups in the TCGA cohort. RSEM, RNA-Seq by Expectation-Maximization. Normal group, paired normal tissues.
