# Supplementary figures and images for: Kinase–substrate Edge Biomarkers Provide a More Accurate Prognostic Prediction in ER-negative Breast Cancer
Source: Genomics Proteomics Bioinformatics. 2021 Jan 13;18(5):525–38. doi: 10.1016/j.gpb.2019.11.012 (PMC8377385; doi:10.1016/j.gpb.2019.11.012)

A Stages I + II

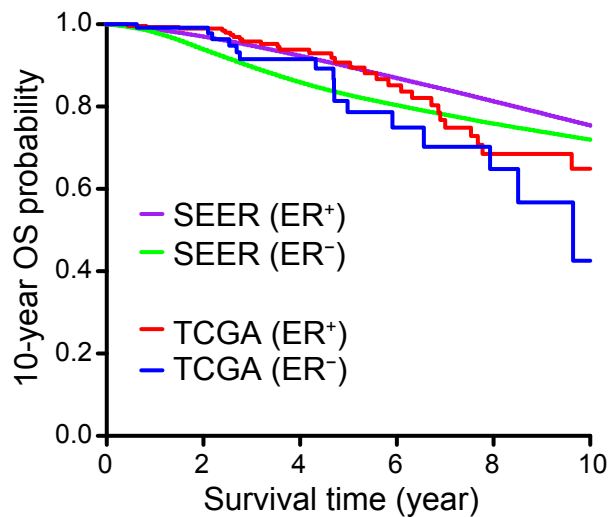

B Stages III + IV

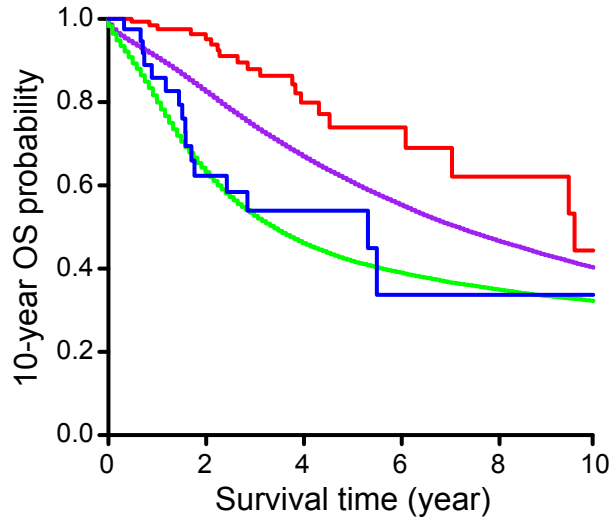

Supplement: Supplementary Figure S1 — OS probability of breast cancer patients at different stages. The 10-year OS probability of ER-positive and ER-negative patients with stages I+II (SEER, P < 0.001; TCGA, P = 0.326; Log-rank test) (A) or stages III+IV (SEER, P < 0.001; TCGA, P < 0.001; Log-rank test) (B). OS, overall survival. [file mmc1.pdf]

**A** Patients aged < 50 years

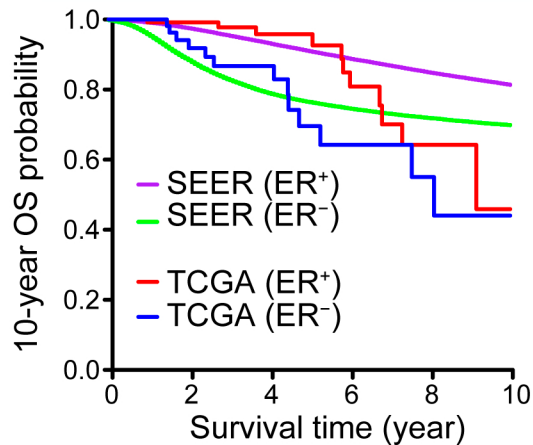

**B** Patients aged 50–69 years

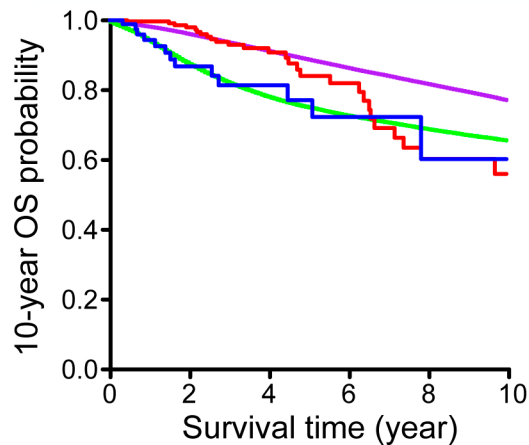

**C** Patients aged ≥ 70 years

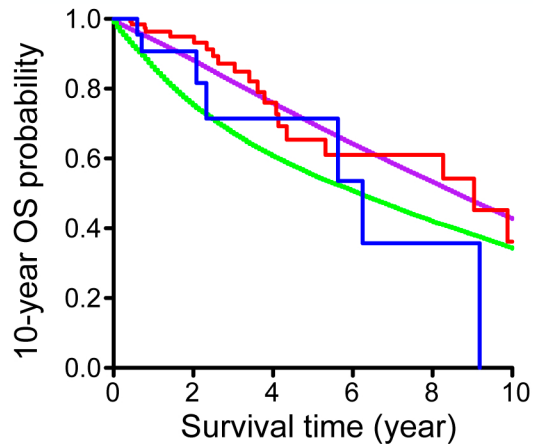

Supplement: Supplementary Figure S2 — OS probability of breast cancer patients in different age groups. The 10-year OS probability of ER-positive and ER-negative patients aged <50 years (SEER, P < 0.001; TCGA, P = 0.049; Log-rank test) (A), 50–69 years (SEER, P < 0.001; TCGA, P = 0.227; Log-rank test) (B), and ≥ 70 years (SEER, P < 0.001; TCGA, P = 0.120; Log-rank test) (C). [file mmc2.pdf]

A Positive lymph node status

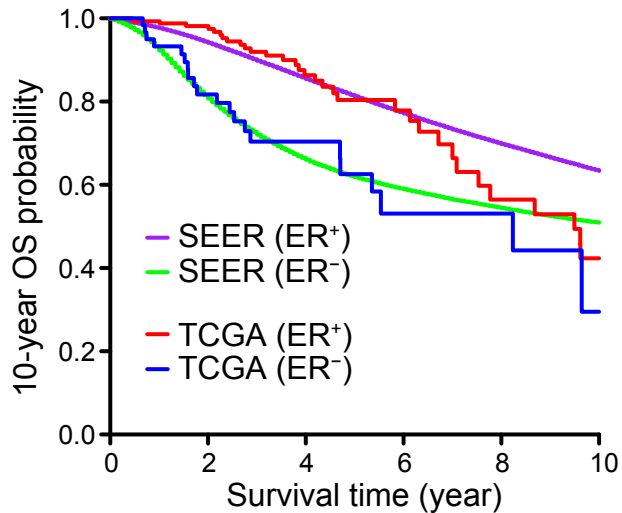

B Negative lymph node status

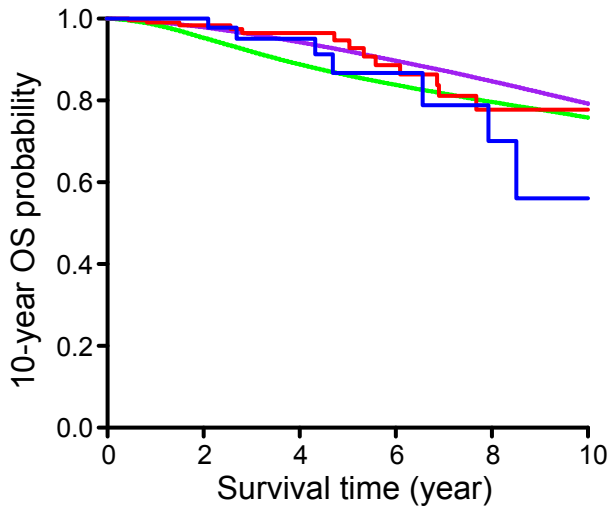

Supplement: Supplementary Figure S3 — OS probability of breast cancer patients with different lymph node status. The OS probability of ER-positive and ER-negative patients with positive lymph node status (SEER, P < 0.001; TCGA, P = 0.007; Log-rank test) (A), or negative lymph node status (SEER, P < 0.001; TCGA, P = 0.778; Log-rank test) (B). [file mmc3.pdf]

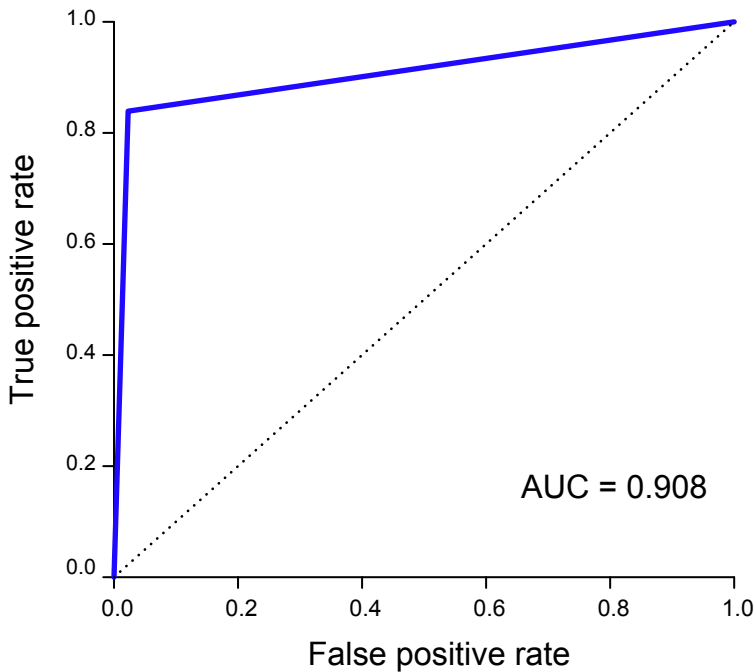

Supplement: Supplementary Figure S4 — ROC curve of the binary classifier against ER status trained with LASSO through five-fold cross validation. [file mmc4.pdf]

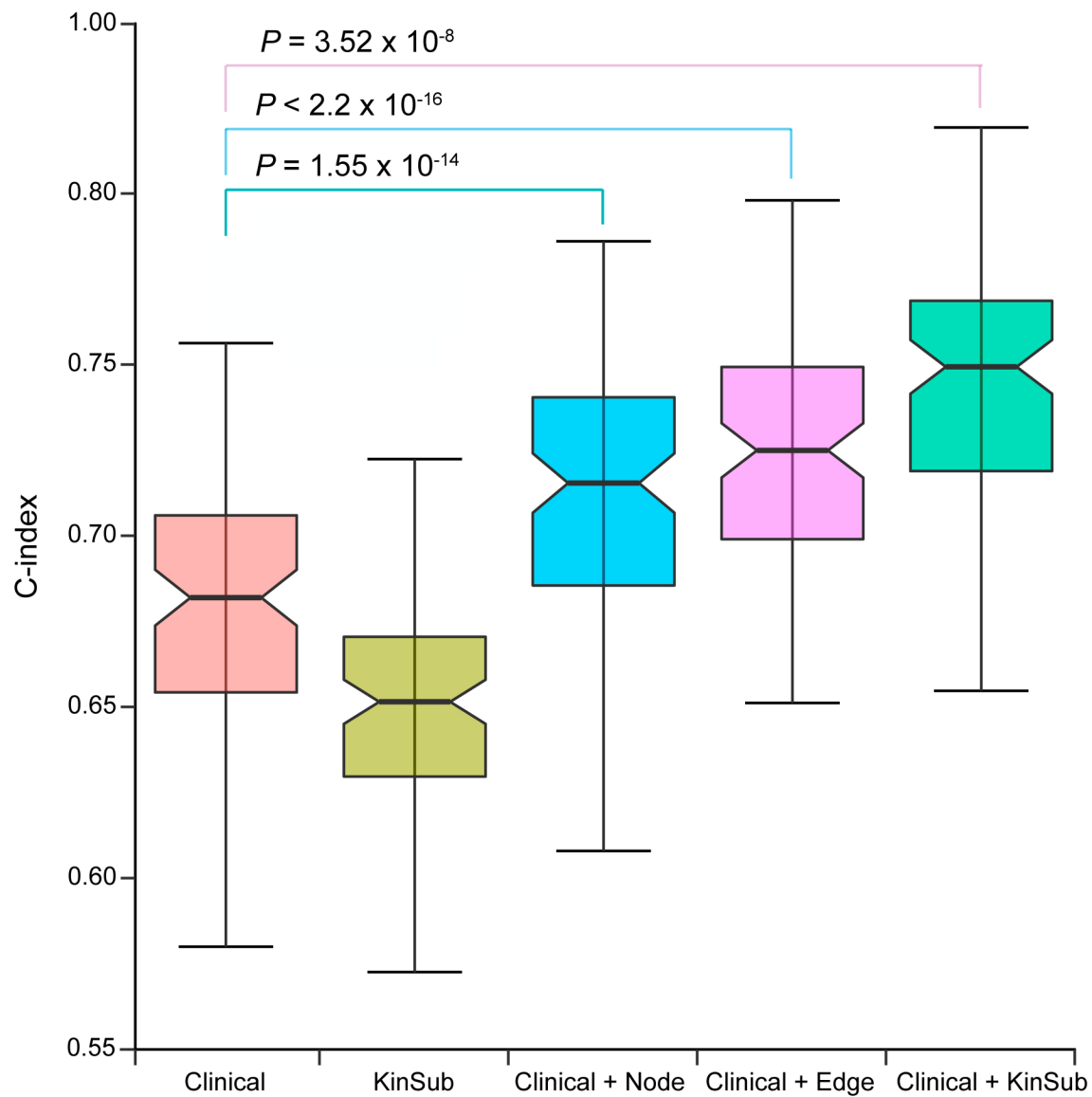

Supplement: Supplementary Figure S5 — Random survival forest models trained from clinical variables and kinase–substrate node and edge features with two-fold cross validation. [file mmc5.pdf]

$$P = 8.86 \times 10^{-6}$$

$$P = 5.89 \times 10^{-14}$$

$$P = 0.021$$

$$P = 2.73 \times 10^{-4}$$

$$P = 3.46 \times 10^{-5}$$

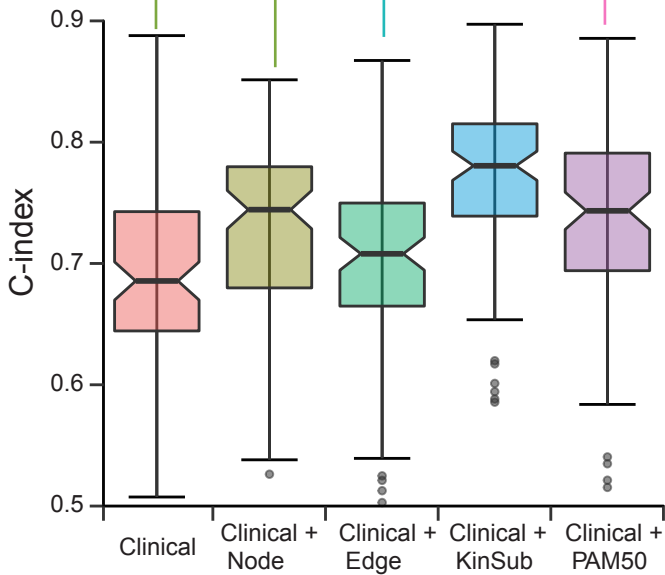

Supplement: Supplementary Figure S6 — C-indexes of 100 times of cross validation of random survival forest models in TCGA dataset. PAM50, 50-gene qPCR assay, which was used to define four intrinsic subtypes of breast cancer. [file mmc6.pdf]

**A TCGA**

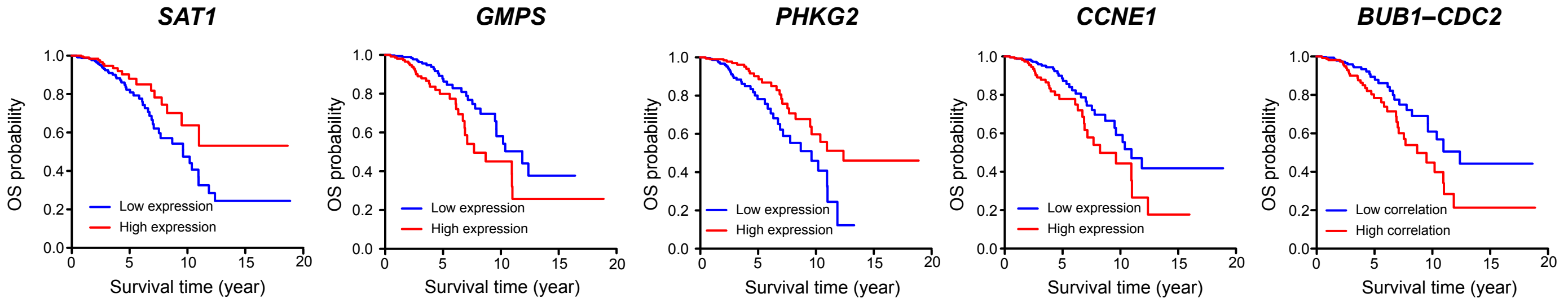

**B GSE42568**

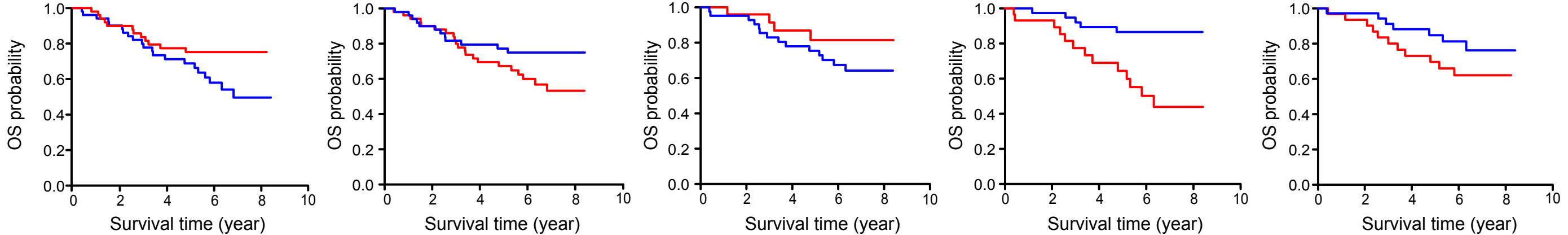

**C GSE25055**

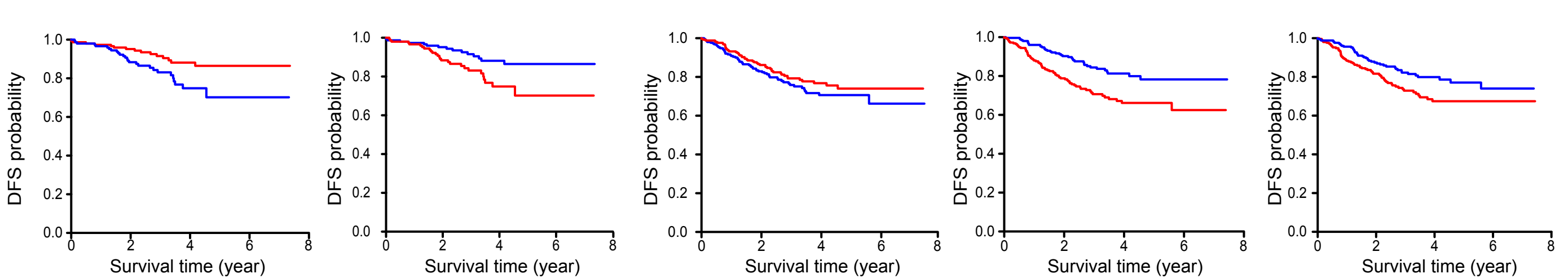

Supplement: Supplementary Figure S7 — Prognostic values of node and edge biomarkers in ER-positive patients A. Kaplan–Meier curves of ER-positive breast cancer patients indicating the prognostic values of the five biomarkers in TCGA cohort. Log-rank test; P = 0.024 for SAT1, P = 0.01 for GMPS, P = 0.005 for PHKG2, P = 0.014 for CCNE1, and P = 0.012 for BUB1–CDC20. B. Kaplan–Meier curves of ER-positive breast cancer patients indicating the prognostic values of the five biomarkers in GSE42568 dataset. Log-rank test; P = 0.057 for SAT1, P = 0.107 for GMPS, P = 0.22 for PHKG2, P = 0.001 for CCNE1, and P = 0.162 for BUB1–CDC20. C. Kaplan–Meier curves of ER-positive breast cancer patients indicating the prognostic values of the five biomarkers in GSE25055 dataset. Log-rank test; P = 0.015 for SAT1, P = 0.015 for GMPS, P = 0.225 for PHKG2, P < 0.001 for CCNE1, and P = 0.016 for BUB1–CDC20. DFS, disease-free survival. [file mmc7.pdf]

**A ER<sup>+</sup>**

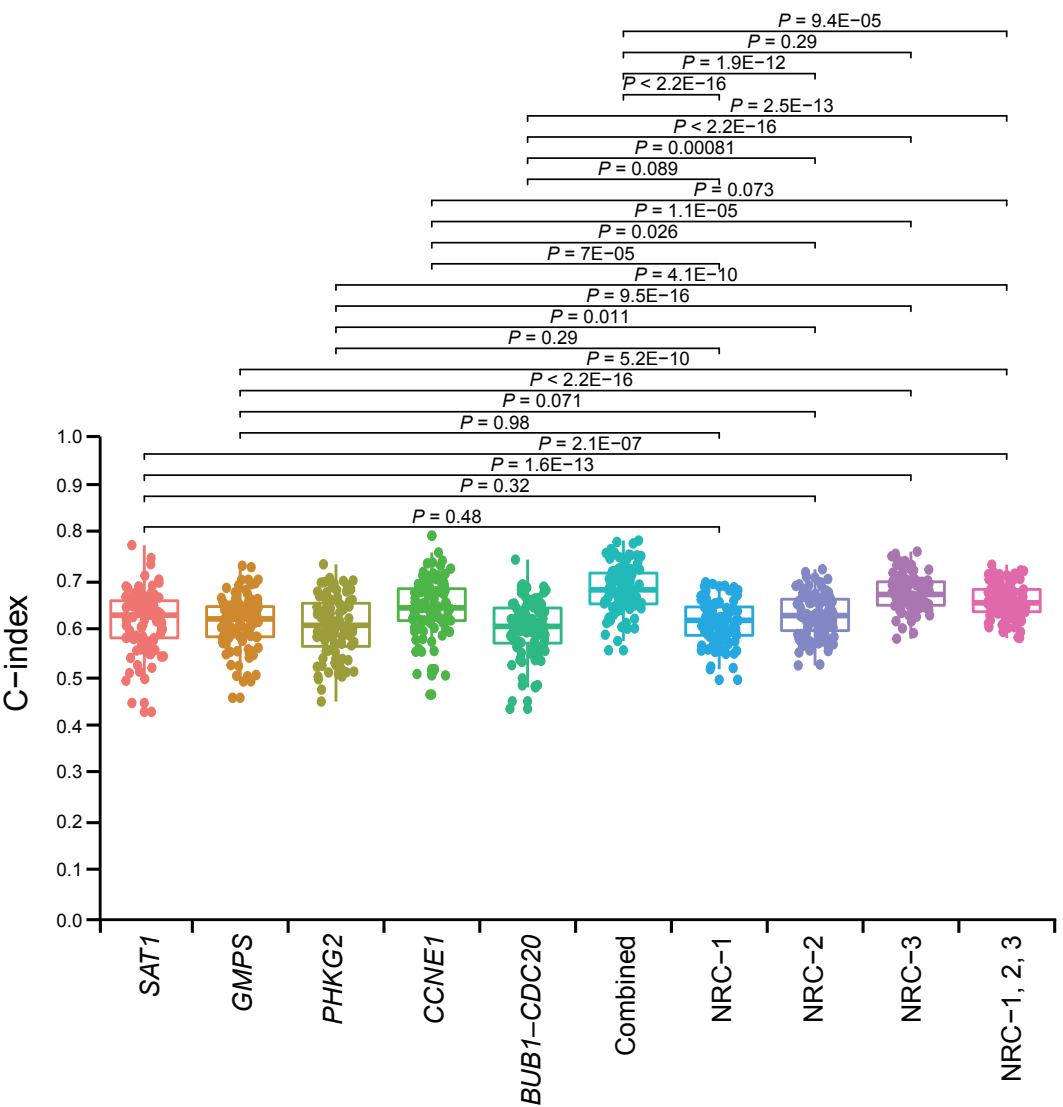

**B ER<sup>-</sup>**

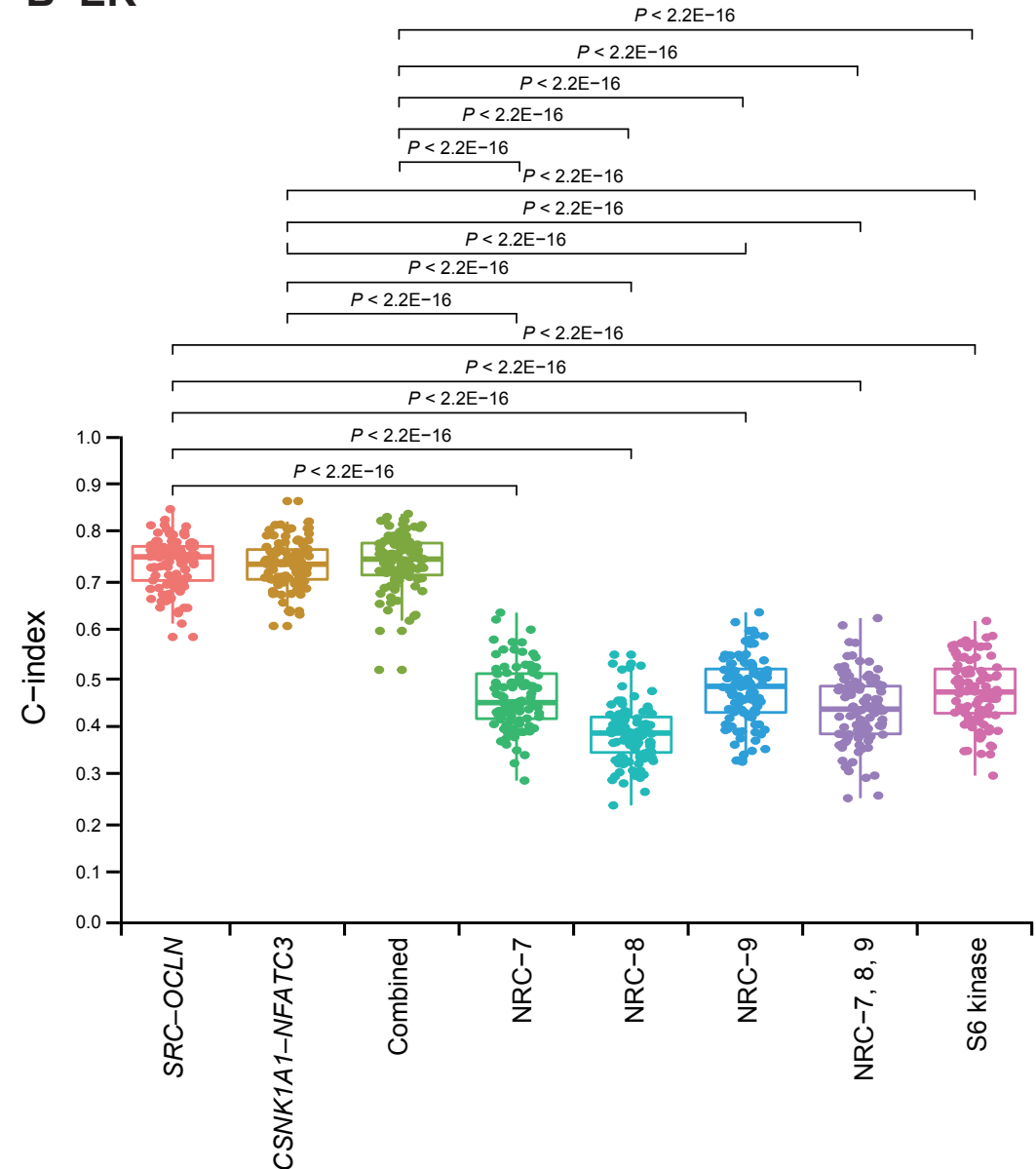

Supplement: Supplementary Figure S8 — Comparison of the kinase–substrate biomarkers with existing biomarkers from previous studies. A. Comparison of prognostic values of biomarkers identified in this study for ER-positive breast cancer and NRC-1, NRC-2, and NRC-3 from Li and colleagues [37]. Combined, the combination of SAT1, GMPS, PHKG2, CCNE1 and BUB1–CDC20. B. Comparison of prognostic values of biomarkers identified in this study for ER-negative breast cancer and NRC-7, NRC-8, and NRC-9 from Li et al. [37] and the “S6 kinase” markers from Speers and colleagues [12]. Combined, the combination of SRC–OCLN and CSNK1A1–NFATC3. NRC, National Research Council gene signatures. [file mmc8.pdf]

**A**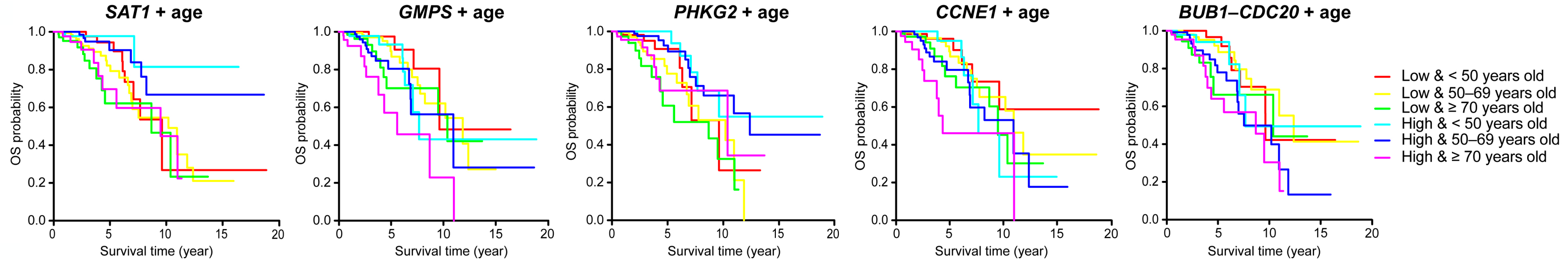**B**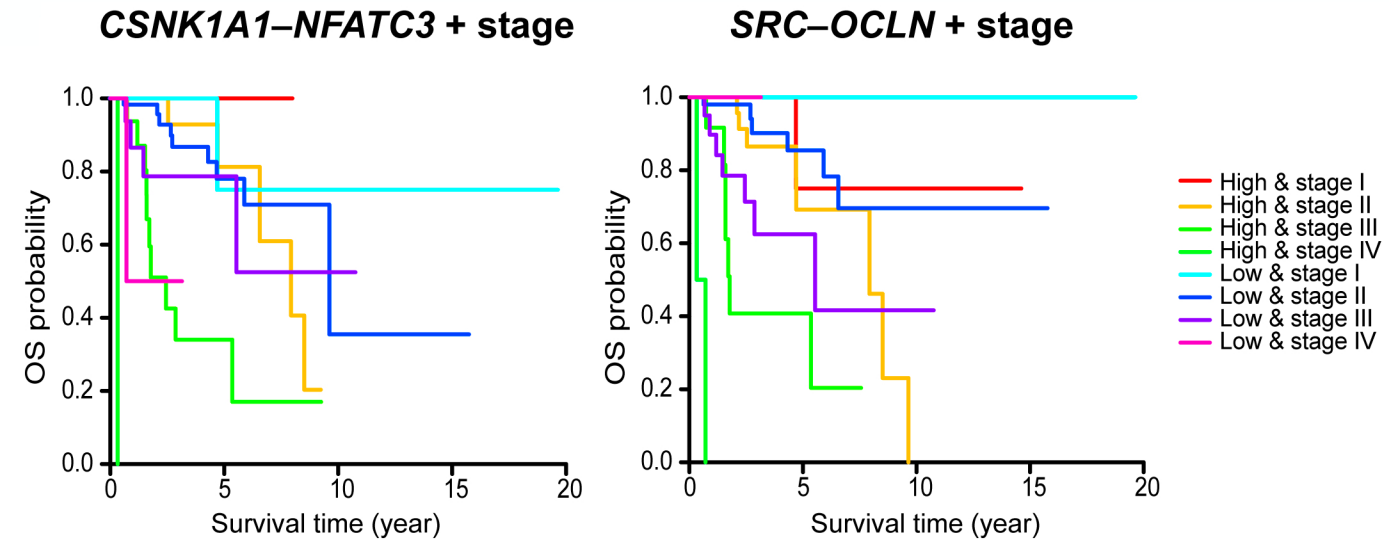**C** ***CSNK1A1-NFATC3* + lymph node status** ***SRC-OCLN* + lymph node status**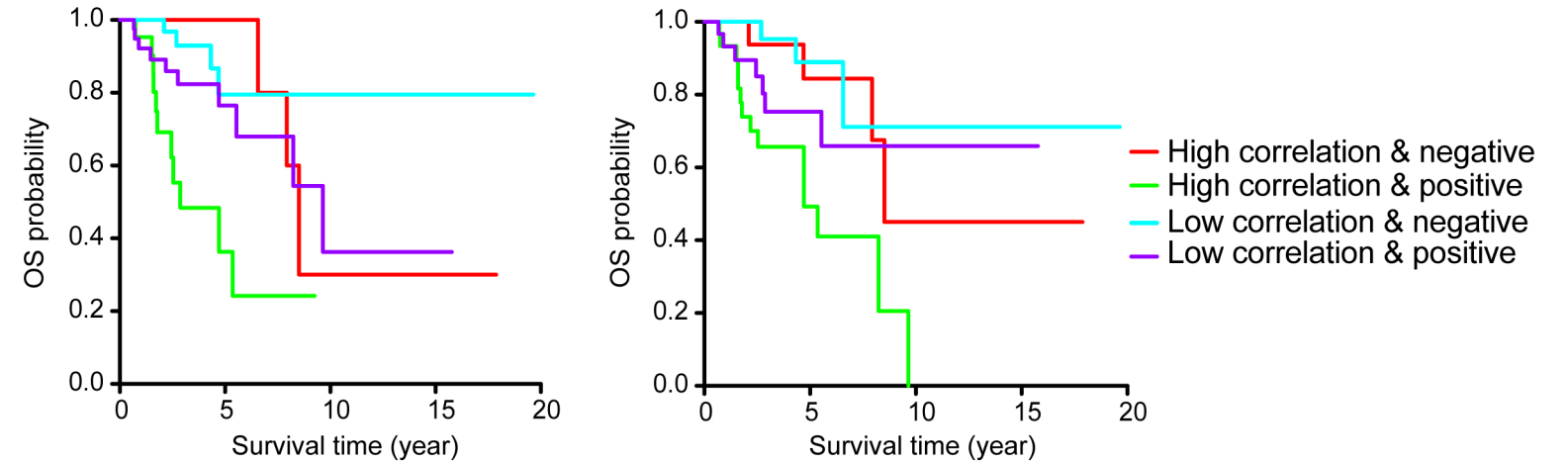

Supplement: Supplementary Figure S9 — Prognostic power integrating the kinase–substrate biomarkers with age group, AJCC stage, or lymph node status. A. Kaplan–Meier curves of ER-positive breast cancer patients indicating the prognostic power of ER-positive biomarkers combined with age groups. P = 0.01 for SAT1 + age, P < 0.001 for GMPS + age, P = 0.005 for PHKG2 + age, P < 0.001 for CCNE1 + age, and P = 0.012 for BUB1–CDC20 + age. B. Kaplan–Meier curves of ER-negative breast cancer patients indicating the prognostic power of ER-negative biomarkers combined with AJCC stages. P < 0.001 for CSNK1A1–NFATC3+ stage and P < 0.001 for SRC–OCLN+ stage. C. Kaplan–Meier curves of ER-negative breast cancer patients indicating the prognostic power of ER-negative biomarkers combined with lymph node statuses. P < 0.001 for CSNK1A1–NFATC3 + lymph node status and P < 0.001 for SRC–OCLN + lymph node status. P values were calculated by Log-rank test. [file mmc9.pdf]
