# Supplementary material for: Kinase–substrate Edge Biomarkers Provide a More Accurate Prognostic Prediction in ER-negative Breast Cancer
Source: Genomics Proteomics Bioinformatics. 2021 Jan 13;18(5):525–38. doi: 10.1016/j.gpb.2019.11.012 (PMC8377385; doi:10.1016/j.gpb.2019.11.012)
Supplement: Supplementary Figure S10 — Expression of the kinases and substrates constructing the ER-positive and ER-negative biomarkers. A. Expression of kinases and substrates of ER-positive biomarkers in normal, ER-positive, and ER-negative groups in the TCGA cohort. B. Expression of kinases and substrates of ER-negative biomarkers in normal, ER-positive, and ER-negative groups in the TCGA cohort. RSEM, RNA-Seq by Expectation-Maximization. Normal group, paired normal tissues. [file mmc10.pdf]

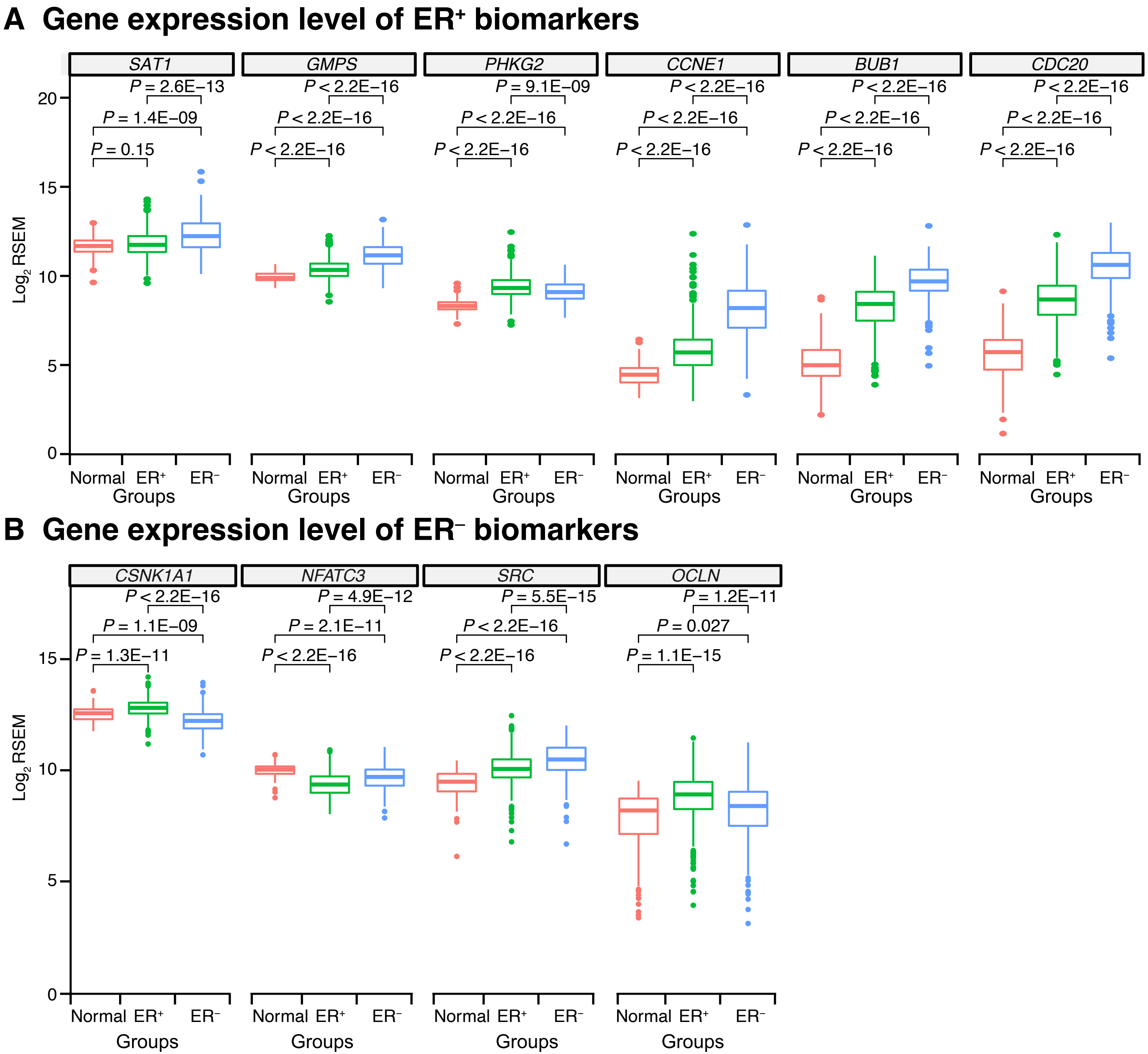
A Gene expression level of ER+ biomarkers
SAT1
GMPS
PHKG2
CCNE1
BUB1
CDC20
P = 2.6E−13
P = 1.4E−09
P = 0.15
P < 2.2E−16
P = 9.1E−09
Log2 RSEM
Normal ER+ ER−
Groups
B Gene expression level of ER− biomarkers
CSNK1A1
NFATC3
SRC
OCLN
P < 2.2E−16
P = 1.1E−09
P = 1.3E−11
P = 4.9E−12
P = 2.1E−11
P = 5.5E−15
P = 1.2E−11
P = 0.027
P = 1.1E−15
Log2 RSEM
Normal ER+ ER−
Groups
