# Supplementary material for: Kinase–substrate Edge Biomarkers Provide a More Accurate Prognostic Prediction in ER-negative Breast Cancer
Source: Genomics Proteomics Bioinformatics. 2021 Jan 13;18(5):525–38. doi: 10.1016/j.gpb.2019.11.012 (PMC8377385; doi:10.1016/j.gpb.2019.11.012)
Supplement: Supplementary Table S1 [file mmc11.docx]

**Table S1 Five-year survival rates for breast cancer patients with four AJCC stages and three age groups**

|  | **SEER (%)** | | |  | **TCGA (%)** | | |
| --- | --- | --- | --- | --- | --- | --- | --- |
|  | **ER^+^** | **ER^−^** | ***P* value** |  | **ER^+^** | **ER^−^** | ***P* value** |
| **All stages** | 84.3 ± 0.006 | 72.2 ± 0.126 | < 0.001 |  | 84.9 ± 2.606 | 72.9 ± 5.160 | 0.02 |
| I | 91.7 ± 0.006 | 88.9 ± 0.156 | 0.173 |  | 93.0 ± 3.510 | 71.4 ± 17.100 | 0.305 |
| II | 86.4 ± 0.009 | 77.3 ± 0.193 | < 0.001 |  | 89.6 ± 3.180 | 79.6 ± 6.400 | 0.122 |
| III | 71.6 ± 0.201 | 50.4 ± 0.333 | < 0.001 |  | 78.1 ± 6.670 | 55.3 ± 9.920 | < 0.001 |
| IV | 26.0 ± 0.360 | 11.7 ± 0.413 | < 0.001 |  | 46.2 ± 16.700 | 33.3 ± 27.200 | 0.132 |
| **All ages** | 84.3 ± 0.006 | 72.2 ± 0.126 | < 0.001 |  | 84.9 ± 2.606 | 72.9 ± 5.160 | 0.018 |
| < 50 years | 91.5 ± 0.090 | 76.9 ± 0.205 | < 0.001 |  | 95.8 ± 2.512 | 69.6 ± 8.750 | 0.048 |
| 50–69 years | 89.3 ± 0.068 | 75.6 ± 0.177 | < 0.001 |  | 85.9 ± 3.685 | 77.1 ± 6.730 | 0.216 |
| ≥ 70 years | 71.2 ± 0.125 | 56.4 ± 0.313 | < 0.001 |  | 65.4 ± 7.960 | 71.4 ± 13.120 | 0.111 |

*Note*: ER, estrogen receptor; SEER, the Surveillance, Epidemiology, and End Results; TCGA, the Cancer Genome Atlas. Data are presented as mean ± SD. Log-rank test.
