# Supplementary material for: Kinase–substrate Edge Biomarkers Provide a More Accurate Prognostic Prediction in ER-negative Breast Cancer
Source: Genomics Proteomics Bioinformatics. 2021 Jan 13;18(5):525–38. doi: 10.1016/j.gpb.2019.11.012 (PMC8377385; doi:10.1016/j.gpb.2019.11.012)
Supplement: Supplementary Table S2 [file mmc12.docx]

**Table S2 Univariate survival analysis of clinical factors in breast cancer patients**

|  | **SEER** | |  | **TCGA** | |
| --- | --- | --- | --- | --- | --- |
|  | **HR (95% CI)** | ***P* value** |  | **HR (95% CI)** | ***P* value** |
| **Histological type (ER^−^ *vs*. ER^+^)** | 1.372 (1.358–1.386) | < 0.001 |  | 1.642 (1.078–2.501) | 0.021 |
| **Age (*vs*. < 50 years)** |  |  |  |  |  |
| 50–69 years | 1.265 (1.247–1.282) | < 0.001 |  | 1.144 (0.7–1.87) | 0.592 |
| ≥ 70 years | 3.675 (3.626–3.724) | < 0.001 |  | 2.191 (1.274–3.766) | 0.005 |
| **Race (*vs*. Caucasian)** |  |  |  |  |  |
| African American | 1.446 (1.426–1.467) | < 0.001 |  | 1.073 (0.647–1.780) | 0.785 |
| American Indian/Alaska Native | 1.105 (1.038–1.177) | 0.002 |  | 0 (0–Inf) | 0.996 |
| Asian or Pacific Islander | 0.683 (0.669–0.697) | < 0.001 |  | 0.415 (0.057–2.991) | 0.383 |
| Unknown | 0.618 (0.559–0.683) | < 0.001 |  | 1.453 (0.575–3.671) | 0.43 |
| **AJCC stage (*vs*. I)** |  |  |  |  |  |
| II | 1.467 (1.45–1.484) | < 0.001 |  | 1.125 (0.6–2.108) | 0.713 |
| III | 3.19 (3.149–3.232) | < 0.001 |  | 2.481 (1.289–4.776) | 0.007 |
| IV | 13.528 (13.31–13.749) | < 0.001 |  | 5.304 (2.194–12.818) | < 0.001 |
| Unknown | 2.606 (2.56–2.654) | < 0.001 |  | 2.657 (1.095–6.445) | 0.031 |
| **Nodal status (*vs*. positive)** |  |  |  |  |  |
| Negative | 2.053 (2.031–2.075) | < 0.001 |  | 2.379 (1.483–3.816) | < 0.001 |
| Unknown | 4.828 (4.772–4.885) | < 0.001 |  | 4.395 (2.327–8.303) | < 0.001 |
| **Tumor grade (*vs*. I)** |  |  |  |  |  |
| II (moderately differentiated) | 1.371 (1.35–1.392) | < 0.001 |  |  |  |
| III (poorly differentiated) | 1.952 (1.923–1.982) | < 0.001 |  |  |  |
| IV (undifferentiated) | 1.913 (185–1.977) | < 0.001 |  |  |  |
| Unknown | 1.973 (1.938–2.008) | < 0.001 |  |  |  |
| **Tumor size (*vs*. < 2.0 cm)** |  |  |  |  |  |
| 2–10 cm | 2.039 (2.019–2.059) | < 0.001 |  |  |  |
| Other or unknown | 3.457 (3.404–3.51) | < 0.001 |  |  |  |

*Note*: AJCC, American Joint Committee on Cancer; ER, estrogen receptor; SEER, the Surveillance, Epidemiology, and End Results; TCGA, The Cancer Genome Atlas. Wald test was used for *P* value calculation.
