# Supplementary material for: Kinase–substrate Edge Biomarkers Provide a More Accurate Prognostic Prediction in ER-negative Breast Cancer
Source: Genomics Proteomics Bioinformatics. 2021 Jan 13;18(5):525–38. doi: 10.1016/j.gpb.2019.11.012 (PMC8377385; doi:10.1016/j.gpb.2019.11.012)
Supplement: Supplementary Table S3 [file mmc13.docx]

**Table S3 Multivariate survival analysis of clinical characteristics in breast cancer patients**

|  | **SEER** | |  | **TCGA** | |
| --- | --- | --- | --- | --- | --- |
| **Variable** | **HR (95% CI)** | ***P* value** |  | **HR (95% CI)** | ***P* value** |
| **Histological type (ER^−^ *vs*. ER^+^)** | 1.356 (1.337–1.376) | < 0.001 |  | 2.170 (1.330–3.541) | 0.002 |
| **Age (*vs*. < 50 years)** |  |  |  |  |  |
| 50$-$69 years | 1.479 (1.455–1.505) | < 0.001 |  | 1.251 (0.733–2.315) | 0.412 |
| ≥ 70 years | 4.785 (4.705–4.866) | < 0.001 |  | 2.373 (1.254–4.488) | 0.008 |
| **Race (*vs*. Caucasian)** |  |  |  |  |  |
| African American | 1.364 (1.338–1.389) | < 0.001 |  | 0.981 (0.543–1.773) | 0.949 |
| American Indian/Alaska Native | 1.329 (1.230–1.436) | < 0.001 |  | – | 0.996 |
| Asian or Pacific Islander | 0.784 (0.764–0.805) | < 0.001 |  | 0.75 (0.102–5.527) | 0.778 |
| **AJCC stage (*vs*. I)** |  |  |  |  |  |
| II | 1.008 (0.986–1.031) | 0.46 |  | 0.952 (0.442–2.05) | 0.901 |
| III | 1.913 (1.858–1.969) | < 0.001 |  | 1.633 (0.645–4.137) | 0.301 |
| IV | 6.152 (5.924–6.390) | < 0.001 |  | 3.241 (0.95–11.061) | 0.06 |
| **Nodal status (Negative *vs*. Positive)** | 1.377 (1.352–1.403) | < 0.001 |  | 1.839 (0.964–3.509) | 0.064 |
| **Tumor grade (*vs*. I)** |  |  |  | – | – |
| II (moderately differentiated) | 1.190 (1.168–1.213) | < 0.001 |  | – | – |
| III (poorly differentiated) | 1.479 (1.450–1.508) | < 0.001 |  | – | – |
| IV (undifferentiated) | 1.438 (1.376–1.493) | < 0.001 |  | – | – |
| **Tumor size (< 2.0 cm *vs*. 2–10 cm)** | 1.516 (1.491–1.541) | < 0.001 |  | – | – |

*Note*: AJCC, American Joint Committee on Cancer; CI, confidence interval; ER, estrogen receptor; HR, hazard ratio; SEER, the Surveillance, Epidemiology, and End Results; TCGA, The Cancer Genome Atlas. Wald test was used for *P* value calculation.
