# Supplementary material for: Kinase–substrate Edge Biomarkers Provide a More Accurate Prognostic Prediction in ER-negative Breast Cancer
Source: Genomics Proteomics Bioinformatics. 2021 Jan 13;18(5):525–38. doi: 10.1016/j.gpb.2019.11.012 (PMC8377385; doi:10.1016/j.gpb.2019.11.012)
Supplement: Supplementary Table S4 [file mmc14.docx]

**Table S4 Multivariate survival analysis of prominent prognostic kinase–substrate biomarkers and clinical factors in ER^+^ and ER^−^ breast cancers**

|  |  | **HR (95% CI)** | ***P* value** |
| --- | --- | --- | --- |
| ER^+^ | Age (*vs*. < 50 years) | 1.552 (0.734–3.284) | 0.250 |
|  | 50–69 years | 4.072 (1.737–9.549) | 0.001 |
|  | ≥ 70 years | 0.997 (0.468–2.127) | 0.994 |
|  | Lymph node status (positive *vs*. negative) | 3.389 (1.534–7.486) | 0.003 |
|  | *SAT1* (high expression *vs*. low expression) | 0.432 (0.218–0.858) | 0.017 |
|  | *GMPS* (high expression *vs*. low expression) | 1.311 (0.608–2.824) | 0.049 |
|  | *PHKG2* (high expression *vs*. low expression) | 0.404 (0.220–0.743) | 0.004 |
|  | *CCNE1* (high expression *vs*. low expression) | 1.770 (0.861–3.637) | 0.009 |
|  | *BUB1*–*CDC20* (high correlation *vs*. low correlation) | 2.164 (1.142–4.100) | 0.018 |
| ER**^−^** | Age (*vs*. < 50 years) | 0.711 (0.296–1.71) | 0.447 |
|  | 50–69 years | 1.531 (0.516–4.542) | 0.443 |
|  | ≥ 70 years | 2.843 (1.039–7.782) | 0.042 |
|  | Lymph node status (positive *vs*. negative) | 1.823 (0.599–5.547) | 0.29 |
|  | *CSNK1A1*–*NFATC3* (high correlation *vs*. low correlation ) | 2.24 (0.999–5.022) | 0.05 |
|  | *SRC*–*OCLN* (high correlation *vs*. low correlation ) | 3.502 (1.535–7.99) | 0.003 |

*Note*: CI, confidence interval; HR, hazard ratio. Wald test was used for *P* value calculation.
