# Supplementary material for: Kinase–substrate Edge Biomarkers Provide a More Accurate Prognostic Prediction in ER-negative Breast Cancer
Source: Genomics Proteomics Bioinformatics. 2021 Jan 13;18(5):525–38. doi: 10.1016/j.gpb.2019.11.012 (PMC8377385; doi:10.1016/j.gpb.2019.11.012)
Supplement: Supplementary Table S5 [file mmc15.pdf]

Table S5 Prognostic values of the kinase–substrate node and edge biomarkers in ten independent GEO datasets

Dataset	ER status (patient No.)	Biomarker	5-year overall survival rate (%)		*P* value (Log-rank test)	HR (95% CI)	Cox *P* value (likelihood test)
			Low expression/ correlation	High expression/ correlation			
GSE10893	ER^+ (n = 71)	*SAT1*	–	–	–	–	–
		GMPS	83.2±11.5	0±0	0.016*	5.999 (1.152–31.255)	0.023*
		PHKG2	46.7±20.7	93.7±4.4	0.034*	0.256 (0.066–0.996)	0.038*
		CCNE1	92.2±5.4	57.6±18.3	0.199	2.412 (0.602–9.669)	0.198
		BUB1–CDC20	91.1±6.4	37.2±26.8	0.057^+	3.288 (0.904–11.952)	0.068**
	ER^- (n = 51)	*CSNK1A1–NFATC3*	70.3±11.2	45.1±13	0.4	1.555 (0.547–4.424)	0.395
		SRC–OCLN	80.1±9.1	34.9±12.6	0.04*	3.04 (0.989–9.344)	0.036*
GSE2034	ER^+ (n = 209)	*SAT1*	62.4±4.6	73.1±4.5	0.127	0.706 (0.451–1.104)	0.123
		GMPS	71.4±4	61.3±5.4	0.13	1.402 (0.903–2.176)	0.135
		PHKG2	60.1±4.8	74.4±4.2	0.022*	0.598 (0.383–0.933)	0.022*
		CCNE1	74.7±3.9	56±5.5	0.005*	1.847 (1.191–2.864)	0.007*
		BUB1–CDC20	76.2±3.9	55±5.3	0.001*	1.608 (1.357–2.092)	0.001*
	ER^- (n = 77)	*CSNK1A1–NFATC3*	73.2±6.9	55.6±8.3	0.075^+	1.845 (0.856–3.977)	0.079^+
		SRC–OCLN	78.4±6.8	52.5±7.9	0.016*	2.659 (1.163–6.08)	0.015*
GSE21653	ER^+ (n = 140)	*SAT1*	73.2±5.5	75.7±5.7	0.56	0.835 (0.455–1.531)	0.559
		GMPS	79.5±4.4	64.3±7.6	0.049*	1.839 (0.993–3.407)	0.059**
		PHKG2	66.7±6.1	81.5±4.8	0.13	0.63 (0.345–1.151)	0.131
		CCNE1	82.5±4	52.3±8.8	<0.001*	2.813 (1.537–5.149)	0.001*
		BUB1–CDC20	81±4.3	60.8±7.8	0.022*	1.996 (1.091–3.65)	0.028*
	ER^- (n = 110)	*CSNK1A1–NFATC3*	72.6±6.9	50.3±7.4	0.041*	1.948 (1.014–3.741)	0.04*
		SRC–OCLN	79.4±6.2	43.3±7.7	<0.001*	3.87 (1.868–8.017)	<0.001*
GSE22133	ER^+ (n = 222)	*SAT1*	79.6±3.8	84.7±3.5	0.038*	0.647 (0.428–0.98)	0.038*
		GMPS	–	–	–	–	–
		PHKG2	79.2±3.7	85.6±3.6	0.041*	0.434 (0.016–0.852)	0.04*
		CCNE1	88.6±2.7	70.2±5.2	0.121	1.388 (0.916–2.103)	0.127
		BUB1–CDC20	–	–	–	–	–
	ER^- (n = 118)	*CSNK1A1–NFATC3*	–	–	–	–	–
		SRC–OCLN	–	–	–	–	–

Dataset	ER status (patient No.)	Biomarker	5-year overall survival rate (%)		*P* value (Log-rank test)	HR (95% CI)	Cox *P* value (likelihood test)
			Low expression/ correlation	High expression/ correlation			
GSE22219	ER^+ (n = 134)	*SAT1*	–	–	–	–	–
		GMPS	87.3±3.7	70.9±6.1	0.001*	2.583 (1.46–4.571)	0.001*
		PHKG2	70.8±5.6	89.9±3.6	0.044*	0.561 (0.317–0.991)	0.044*
		CCNE1	86.7±3.7	70.6±6.4	0.002*	2.337 (1.33–4.108)	0.003*
		BUB1–CDC20	70.1±5.6	91±3.5	<0.001*	0.194 (0.097–0.389)	<0.001*
	ER^- (n = 82)	*CSNK1A1–NFATC3*	85.4±5.5	53.7±7.8	0.007*	1.463 (1.203–2.199)	0.007*
		SRC–OCLN	83.7±5.6	53.8±8	0.029*	1.595 (1.261–2.563)	0.031*
GSE48408	ER^+ (n = 120)	*SAT1*	83.3±4.6	86.8±4.7	0.481	0.829 (0.491–1.398)	0.48
		GMPS	90±3.6	77.6±6	0.041*	1.7 (1.015–2.847)	0.045*
		PHKG2	86±4.6	83.9±4.7	0.637	0.883 (0.528–1.479)	0.637
		CCNE1	89.6±3.5	76.2±6.6	0.127	1.499 (0.888–2.53)	0.136
		BUB1–CDC20	91±3.2	73.2±6.9	0.022*	1.82 (1.081–3.064)	0.027*
	ER^- (n = 44)	*CSNK1A1–NFATC3*	77.8±9.8	50±9.8	0.109	2.045 (0.837–4.995)	0.103
		SRC–OCLN	68.8±11.6	57.1±9.4	0.172	1.894 (0.746–4.81)	0.16
GSE4922	ER^+ (n = 211)	*SAT1*	66.9±4.7	75.7±4.2	0.024*	0.593 (0.374–0.94)	0.024*
		GMPS	78.5±3.8	62±5.2	0.029*	1.645 (1.048–2.581)	0.031*
		PHKG2	69.4±4.7	73±4.3	0.4	0.824 (0.526–1.293)	0.4
		CCNE1	78.4±3.8	62.3±5.1	0.006*	1.875 (1.192–2.949)	0.006*
		BUB1 –CDC20	79.4±3.8	61.5±5.1	<0.001*	1.560 (1.323–2.024)	<0.001*
	ER^- (n = 34)	*CSNK1A1 –NFATC3*	80.1±8.9	40.4±15.5	0.033*	3.307 (1.033–10.585)	0.043*
		SRC –OCLN	84.8±10	51.9±12.4	0.085**	3 (0.804–11.189)	0.079**
GSE53031	ER^+ (n = 124)	*SAT1*	73±6.1	83.9±5.3	0.06^+	0.481 (0.221–1.046)	0.058**
		GMPS	–	–	–	–	–
		PHKG2	71.7±7.1	82.7±4.8	0.107	0.547 (0.26–1.151)	0.115
		CCNE1	82.8±4.8	69.8±7.4	0.065**	1.987 (0.944–4.185)	0.073**
		BUB1 –CDC20	82.8±6	75.2±5.5	0.031*	2.473 (1.055–5.794)	0.026*
	ER^- (n = 43)	*CSNK1A1 –NFATC3*	84.4±8.5	43±13.4	0.012*	4.511 (1.236–16.455)	0.012*
		SRC –OCLN	87.9±8.2	41.5±12.8	0.003*	7.267 (1.603–32.935)	0.002*

Dataset	ER status (patient No.)	Biomarker	5-year overall survival rate (%)		*P* value (Log-rank test)	HR (95% CI)	Cox *P* value (likelihood test)
			Low expression/ correlation	High expression/ correlation			
GSE6532	ER+ (n = 114)	*SAT1*	66.9 ±4.7	75.7 ±4.2	0.024*	0.593 (0.374–0.94)	0.024*
		GMPS	80.7 ±5.2	58.6 ±7	0.006*	2.38 (1.259–4.499)	0.006*
		PHKG2	60.3 ±6.8	79.9 ±5.4	0.019*	0.475 (0.251–0.898)	0.019*
		CCNE1	80 ±5.4	59.9 ±6.8	0.041*	1.911 (1.018–3.587)	0.041*
		BUB1 – CDC20	83.6 ±5	55.8 ±6.9	0.001*	2.905 (1.503–5.616)	0.001*
	ER− (n = 5)	*CSNK1A1 – NFATC3*	NA	NA	NA	NA	NA
		SRC –OCLN	NA	NA	NA	NA	NA
GSE7390	ER+ (n = 134)	*SAT1*	93.1 ±3	94.8 ±2.9	0.132	0.564 (0.265–1.199)	0.126
		GMPS	95.3 ±2.7	92.6 ±3.2	0.036*	2.198 (1.033–4.677)	0.034*
		PHKG2	92.2 ±3.4	95.5 ±2.5	0.173	0.607 (0.294–1.254)	0.171
		CCNE1	97.6 ±1.7	87.7 ±4.7	0.099**	1.795 (0.887–3.635)	0.108
		BUB1 – CDC20	98.8 ±1.2	85.9 ±4.9	0.016*	1.536 (1.235–2.392)	0.019*
	ER− (n = 64)	*CSNK1A1 – NFATC3*	79.2 ±7	70 ±8.4	0.11	1.905 (0.853–4.251)	0.111
		SRC –OCLN	77.9 ±5.9	63.5 ±13.1	0.02*	1.480 (1.189–2.433)	0.034*

Note: CI, confidence interval; ER, estrogen receptor; HR, hazard ratio; NA, values were unavailable as the sample size was too small to calculate; –, the gene was unavailable in the dataset; *, $P < 0.05$; **, $P < 0.1$. Data are presented as mean ± SD.
