# Supplementary material for: Kinase–substrate Edge Biomarkers Provide a More Accurate Prognostic Prediction in ER-negative Breast Cancer
Source: Genomics Proteomics Bioinformatics. 2021 Jan 13;18(5):525–38. doi: 10.1016/j.gpb.2019.11.012 (PMC8377385; doi:10.1016/j.gpb.2019.11.012)
Supplement: Supplementary Table S6 [file mmc16.docx]

**Table S6 Prognostic power of kinase–substrate biomarkers in two breast cancer subtypes considering age group, AJCC stage, or lymph node status**

|  | **Biomarker** | **C-index** | ***P* value** |
| --- | --- | --- | --- |
| ER^+^ | *SAT1* | 0.569 | 0.021 |
|  | *SAT1* + age | 0.674 | 0.001 |
|  | *SAT1* + stage | 0.652 | 0.004 |
|  | *SAT1* + lymph node status | 0.630 | 0.003 |
|  | *GMPS* | 0.608 | 0.016 |
|  | *GMPS* + age | 0.696 | < 0.001 |
|  | *GMPS* + stage | 0.657 | 0.003 |
|  | *GMPS* + lymph node status | 0.645 | 0.001 |
|  | *PHKG2* | 0.585 | 0.004 |
|  | *PHKG2* + age | 0.684 | < 0.001 |
|  | *PHKG2* + stage | 0.654 | 0.001 |
|  | *PHKG2* + lymph node status | 0.628 | 0.001 |
|  | *CCNE1* | 0.577 | 0.019 |
|  | *CCNE1* + age | 0.667 | 0.001 |
|  | *CCNE1* + stage | 0.649 | 0.002 |
|  | *CCNE1* + lymph node status | 0.619 | 0.006 |
|  | *BUB1*–*CDC20* | 0.579 | 0.017 |
|  | *BUB1*–*CDC20* + age | 0.674 | 0.002 |
|  | *BUB1*–*CDC20* + stage | 0.658 | 0.003 |
|  | *BUB1*–*CDC20* + lymph node status | 0.629 | 0.001 |
| ER^−^ | *CSNK1A1*–*NFATC3* | *0.577* | *0.045* |
|  | *CSNK1A1*–*NFATC3 +* age | *0.624* | *0.056* |
|  | *CSNK1A1*–*NFATC3* + stage | 0.793 | < 0.001 |
|  | *CSNK1A1*–*NFATC3* + lymph node status | 0.737 | 0.001 |
|  | *SRC*–*OCLN* | 0.591 | 0.046 |
|  | *SRC*–*OCLN* + age | 0.638 | 0.077 |
|  | *SRC*–*OCLN* + stage | 0.778 | < 0.001 |
|  | *SRC*–*OCLN* + lymph node status | 0.739 | < 0.001 |

*Note*: ER, estrogen receptor. Wald test was used for *P* value calculation.
